# On the role of sex steroids in biological functions by classical and non-classical pathways. An update

**DOI:** 10.1016/j.yfrne.2021.100926

**Published:** 2021-06-03

**Authors:** Miriam Pillerová, Veronika Borbélyová, Július Hodosy, Vladimír Riljak, Emese Renczés, Karyn M. Frick, Ľubomíra Tóthová

**Affiliations:** a Institute of Molecular Biomedicine, Faculty of Medicine, Comenius University in Bratislava, Bratislava, Slovakia; b Institute of Physiology, First Faculty of Medicine, Charles University, Prague, Czech Republic; c Department of Psychology, University of Wisconsin-Milwaukee, Milwaukee, WI, USA

**Keywords:** Sex steroids, Gonadal hormones, Brain structures, Intracellular receptors, Transmembrane receptors, Classical/non-classical signaling

## Abstract

The sex steroid hormones (SSHs) play several roles in regulation of various processes in the cardiovascular, immune, muscular and neural systems. SSHs affect prenatal and postnatal development of various brain structures, including regions associated with important physiological, behavioral, cognitive, and emotional functions. This action can be mediated by either intracellular or transmembrane receptors. While the classical mechanisms of SSHs action are relatively well examined, the physiological importance of non-classical mechanism of SSHs action through membrane-associated and transmembrane receptors in the brain remains unclear. The most recent summary describing the role of SSHs in different body systems is lacking. Therefore, the aim of this review is to discuss classical and non-classical signaling pathways of testosterone and estradiol action via their receptors at functional, cellular, tissue level and to describe the effects on various body systems and behavior. Particular emphasis will be on brain regions including the hippocampus, hypothalamus, frontal cortex and cerebellum.

## 1. Introduction

The sex steroid hormones (SSHs) are known not only as regulators of sexual differentiation, secondary sex characteristics, sexual behaviors, reproduction, but also affect various systems such as skeletal, immune, muscular, and cardiovascular. In addition, SSHs play a pivotal role in brain structure formation and cognitive function (Bhatia et al., 2014; Campbell & Jialal, 2020; Carson & Manolagas, 2015; dos Santos et al., 2014; McEwen & Milner, 2017). Furthermore, SSHs exert pleiotropic effects in the central nervous system promoting neurogenesis and neuroprotection, as well as learning and memory (Diotel et al., 2018; Frick & Kim, 2018; Sun et al., 2019). These effects are mediated not only via intracellular or membrane-associated receptors (such as the androgen receptor (AR), estrogen receptor alpha (ERα), estrogen receptor beta (ERβ)) but also via transmembrane receptors (such as zinc transporter protein 9 (ZIP9), G protein-coupled estrogen receptor 1 (GPER1)). While the classical effects of SSHs via AR, ERα, ERβ are relatively well described, the physiological importance of rapid, non-classical actions of SSHs via membrane-associated (AR, ERα, ERβ) and transmembrane – GPCR steroid receptors (ZIP9, GPER1) is not well understood.

SSHs shape the brain during the critical prenatal and perinatal periods of development (organizational windows) when hormones interact with an immature neural substrate. In this period of life, exposure to SSHs can cause permanent sex differences in brain structures and their functions, which are responsible for the sexual differentiation of the brain and behavior (Cooke et al., 1998; Williams, 1986). Prenatal and perinatal effects of SSHs determine the brain’s response to steroids later in life. In addition, another “organizational window” during the postnatal period of life exists as well – puberty and adolescence (Schulz & Sisk, 2016; Vigil et al., 2016). Besides the organizational effects, SSHs also have activational effects on mature brain structures. Activational effects are acute, reversible and evoke transient behavioral or physiological responses throughout life (Cooke et al., 1998; Williams, 1986). In general, the activational effects of SSHs appear post puberty and act independently or in combination with organizational effects (Schulz & Sisk, 2016). Therefore, both activational and organizational effects of SSHs on the brain could affect behavioral outcomes later in life.

However, a current summary of results of experimental or clinical studies describing the role of testosterone (T) and estrogen (E, mostly estradiol (E_2_)) via classical and non-classical receptors and their role in different body systems is lacking. In this review, we aimed to sum up what is known and update the latest knowledge regarding the role of T and E_2_ in various tissues and body systems with specific focus on the brain via classical and non-classical signaling pathways. First, we discuss the specific effects of T and E (E_2_) via different receptors (AR, ERα, ERβ, ZIP9, GPER1) on various body systems. Subsequently, we describe the role of T and E_2_ in hippocampus (HIP), hypothalamus (HYP), frontal cortex (FC), and cerebellum (CER) – selected brain regions associated with important physiological, behavioral, cognitive, and emotional functions.

## 2. Androgens and estrogens

There are three major classes of sex steroid hormones – androgens, estrogens and progestogens. Androgens are known as primary “male sex hormones” because of their masculinizing effects and Es, together with progestins, as primary “female sex hormones”. However, all of these hormones are synthesized in both, females and males in different concentrations. Testosterone (T), the major androgen, is aromatized to estradiol by the enzyme aromatase and also reduced to the non-aromatizable androgen dihydrotestosterone (DHT) by 5α-reductase (Celotti et al., 1997).

As mentioned previously in the introduction, these hormones play a crucial role throughout the development and exert diverse physiological functions in the body (Bhatia et al., 2014; Campbell & Jialal, 2020; Carson & Manolagas, 2015; Diotel et al., 2018; dos Santos et al., 2014; Frick & Kim, 2018; McEwen & Milner, 2017; Sun et al., 2019). Moreover, SSHs are also implicated in the development of the neurodevelopmental, neurodegenerative, and affective disorders (Crowley, 2017; McHenry et al., 2014; Morrell et al., 2005; Pinares-Garcia et al., 2018; Vadakkadath Meethal & Atwood, 2005). To exert these effects, SSHs must activate the signaling cascade via binding to appropriate intracellular, membrane-associated, or transmembrane receptors.

### 2.1. Receptors for androgens and estrogens

The actions of androgens and estrogens were historically thought to be slow, nuclear processes mediated through hormone receptors located in the cytoplasm complexed to chaperons or in the nucleus, i.e. intracellular AR and ERs (Walters et al., 1981). These processes of intracellular hormone receptors usually result in transcription of specific genes (genomic actions) which may take several hours. For such gene transcriptional responses, the ligand-receptor complex must be localized in the nucleus (Métivier et al., 2003). Later, it has been discovered that ligand-intracellular receptor complexes might be associated with the plasma membrane and can stimulate fast, non-classical processes in the cytoplasm occurring within seconds or minutes (Morley et al., 1992). These actions include various extranuclear downstream cascades regulating different cellular responses, such as DNA synthesis, cell proliferation, migration or survival (Schwartz et al., 2016). Thus, the same intracellular receptor may be responsible for both genomic and rapid responses. The shape and conformational flexibility of the ligands and ligand-binding domains (LBD), e.g. the open/closed position of helix-12, determines whether the accommodated ligand will be agonist/antagonist of the genomic or rapid action (Norman et al., 2001, 2004; Zinn & Schnell, 2018). According to the X-ray structure analysis, there are numerous different LBDs of nuclear steroid-hormone-receptors described in the Protein Data Bank (Bourguet et al., 2000). In several steroid receptors, the presence of a classical and a putative alternative binding site has been identified, mediating the genomic or rapid actions, respectively (Norman et al., 2004). It has been shown that some inter-molecular interactions, such as interactions with a scaffold-proteins or membrane proteins in the caveolae (Fridolfsson et al., 2014), as well as the occupancy of the classical ligand pocket and the absence of the coactivator protein (Bálint et al., 2017) may facilitate conformational changes of the receptor favoring the accommodation of the ligand in the alternative binding site.

Moreover, in the past couple of decades, rapid signaling through 7-transmembrane GPCR receptors, including the androgen receptor ZIP9 and GPER1 has been discovered (Berg et al., 2014; Carmeci et al., 1997; Revankar et al., 2005; Thomas et al., 2014). The role of GPCR receptors are well described in the gonads of both, males and females – apoptosis, spermatogenesis, signaling in Sertoli cells in case of ZIP9, and the proper function of Leydig cells in testes or uterus and reproduction in case of GPER1 (Berg et al., 2014; Kotula-Balak et al., 2018; Olde & Lee, 2009; Thomas et al., 2017a, 2014).

There are numerous studies describing the molecular signaling pathways of these receptors and the effects of SSHs via these receptors on the brain, body and behavior (Table 1). The results of these studies have brought new insights into the neurobehavioral effects of SSHs. On the other hand, additional questions have arisen, such as the sex-, age- and tissue-specific role of rapid, non-classical mechanisms involving the GPCR ZIP9 and GPER1 receptors in the brain.

#### 2.1.1. Intracellular/membrane-associated AR

The binding of T and DHT to nuclear AR was first described in 1968 (Bruchovsky & Wilson, 1968a, 1968b). The expression of AR was detected in various brain areas including HIP, HYP, FC, CER, amygdala and striatum (Mhaouty-Kodja, 2017; Tobiansky et al., 2018b), but also in pancreas (Díaz-Sánchez et al., 1995), prostate (Lee et al., 1995), fibroblasts (Jacobson et al., 1995) or adipose tissue (Dieudonne et al., 1998). The expression of AR was also detected in presumptive pronephros and olfactory placodes of embryos, in pineal organ anlage and retina (3–5 days post-fertilization), and in several other regions of telencephalon, preoptic area and paraventricular nucleus of HYP in adult zebrafishes (Gorelick et al., 2008). The actions via AR signaling pathways are also involved in regulation of many processes in various body systems such as cardiovascular (Ikeda et al., 2005), immune (Gubbels Bupp & Jorgensen, 2018) and hemopoietic systems, glucose and fat metabolism (Lin et al., 2005), prostate epithelial homeostasis (Zhang et al., 2016), bone healing (Komrakova et al., 2020), muscle fast-twitch and hypertrophy (Davey et al., 2017; Morton et al., 2018), and brain masculinization (Sato et al., 2004). Signaling via AR is also involved in prostate (Debes & Tindall, 2002), and breast cancer (Giovannelli et al., 2018), where it promotes the growth of the tissue. Regarding the memory, in intact male mice, the memory consolidation seemed to be protected via AR activation after infusion of aromatase inhibitor letrozole into dorsal HIP in object recognition and object placement tasks in comparison to gonadectomized male mice (Koss & Frick, 2019). The emotional memory, dependent largely on the HIP and amygdala, was tested in the orchiectomized adolescent male rats with or without T or DHT treatment by the inhibitory avoidance test. Orchiectomized rats spent significantly less time in the illuminated box after foot-shock training and had reduced AR-immunoreactivity in amygdala/hippocampal CA1 region in comparison to sham-operated males. The treatment of these male rats with both T and DHT reversed these effects which suggest that androgens enhance inhibitory avoidance memory probably by binding to AR (Islam et al., 2021).

The classical and non-classical signaling pathways via this receptor are activated through androgen hormone ligands, predominantly T and DHT (but also androstenedione, androstenediol, dehydroepiandrosterone) in the cytoplasm (Roy et al., 1999). In the classical signaling cascade, the ligand-receptor complex translocates into the nucleus, where the receptor dimerizes, binds to DNA as a transcription factor together with other proteins, and expresses target genes (Heemers & Tindall, 2007).

**Fig. 1.**
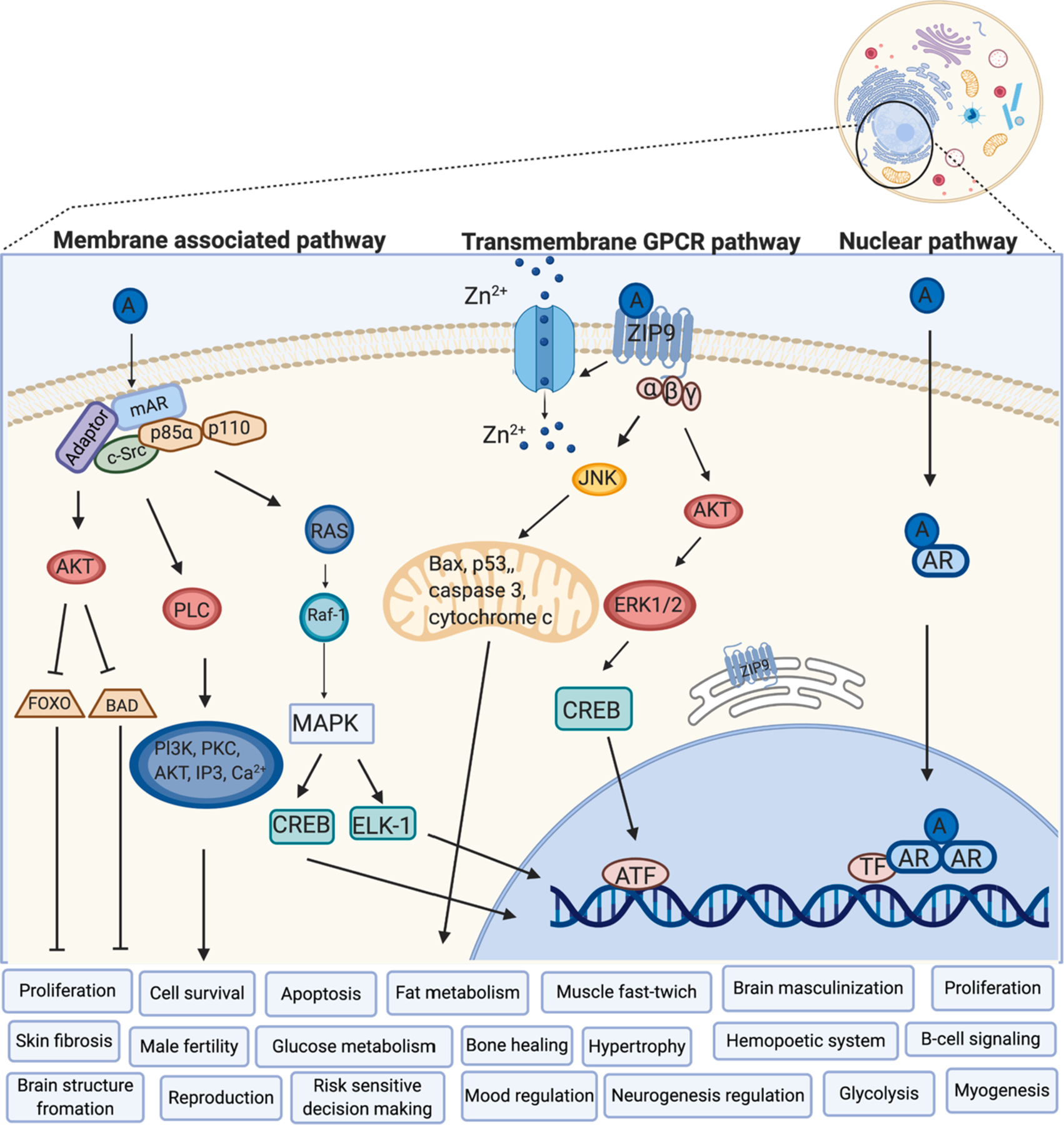
Intracellular, membrane-associated and transmembrane GPCR receptors of androgens. A – Androgen, AR – Androgen Receptor, mAR – membrane-associated Androgen Receptor, ZIP9 – Zinc transporter protein 9, Zrt- and Irt-like protein 9, c-Src – protooncogene tyrosine-protein kinase Src, MAPK – Mitogen-Activated Protein Kinase, ELK-1 – transcription activator, CREB – cAMP Response Element-Binding protein, AKT – protein kinase B, RAS – small GTPases, RAF-1 – proto-oncogene, serine/threonine kinase, FOXO – Forkhead transcription factors of the O class, BAD – Bcl2 Associated Agonist Of Cell Death, PI3K – PhospoInositide 3-Kinase, PLC – PhosphoLipase C, Bax – Bcl2 Associated X, JNK – c-Jun N-terminal kinases, ATF – Activating Transcription Factors, ERK – Extracellular signal-Regulated Kinases, TF – Transcription Factor, Zn^2+^ – Zinc (adapted from (Carrier et al., 2015; Leung & Sadar, 2017; Thomas et al., 2017b) and created with BioRender.com).

Fast, non-classical actions mediated by AR associated with the membrane in a complex with caveolin 1 have been already discovered (Heinlein & Chang, 2002; Lu et al., 2001; Lutz et al., 2003; Papakonstanti et al., 2003). These actions activate second messenger pathways including a) phosphatidylinositol 3 kinase (PI3K) leading to phosphorylation of AKT (known as protein kinase B) in the androgen-sensitive epithelial cells and osteoblasts (Baron et al., 2004; Kang et al., 2004) or activation of b) Src/Shc/ERK (proto-oncogene c-Src/Src homology 2 domain containing/Extracellular Signal-Regulated Kinase) in osteoblasts, osteocytes, embryonic fibroblasts and HeLa cells (Kousteni et al., 2001), and c) MAPK signaling cascade in androgen-sensitive human prostate adenocarcinoma LNCaP cells (Heinlein & Chang, 2002). The non-classical signaling pathway of AR could result in cell survival and cell proliferation through Src/p85α/phosphoinositide 3-kinase further activating MAPK and AKT pathways. ERK is also able to phosphorylate the intracellular AR leading to activation of transcriptional coactivators and transcription itself in the nucleus (Liao et al., 2013). Both, the classical and non-classical signaling of AR are involved in the protective mechanisms in the brains of patients suffering from Alzheimer disease as reviewed in Pike et al. (2008) (Pike et al., 2008). While activation of the classical pathway results in decreased β-amyloid plaques, activation of non-classical signaling pathway led to reduced apoptosis (Pike et al., 2008). The potential signaling mechanisms (intracellular and membrane-associated) via AR and their effects on various body systems are summarized in Fig. 1.

#### 2.1.2. Intracellular/membrane-associated ERs

Intracellular ERs were first identified in 1958 (Jensen, 2012). Their expression was detected in various brain regions such as HIP, HYP, FC, CER, amygdala, olfactory bulb, cerebral cortex, basal forebrain, thalamus, pons Varolii, medulla oblongata, stria terminalis and periventricular preoptic nucleus (Almey et al., 2015; Foster, 2012; Pérez et al., 2003). Within HIP, the expression of ERs was reported in hippocampal pyramidal cells of Ammon’s horn and DG as soon as the 15th gestational week to adulthood. Furthermore, in adulthood, there is more ERβ than ERα in HIP and cerebral cortex (González et al., 2007). In addition, both ERs are expressed in fetal neurons, but only ERα is expressed in the Cajal-Retzius cells of marginal zones (layer I) in developing cerebral cortex and immature hippocampus, which suggest that each of the ERs may play a different role during prenatal development of HIP (González et al., 2007). Regarding other body systems, the expression of ERs was detected in the gastrointestinal tract (Lange & Meyer, 2003), kidney medulla and cortex, liver (Mizutani et al., 1994), lung, spleen, muscles, heart (Lange et al., 2001), mammary gland (Schams, 2003), ovary, uterus, testes, adrenal glands (Hutson et al., 2019) or adipose tissue (Mizutani et al., 1994).

The concentrations of ERs are the highest during the critical or sensitive periods of life (mainly prenatal/neonatal period and puberty), which supports the important role of E in the organization of hippocampal structure during its development (O’Keefe & Handa, 1990). The cellular localization of ERs was detected not only in the neurons but also in glia of the HIP (Mitterling et al., 2010). On the ultrastructural level, 50% of ERα was found in neuronal axons and axon terminals, 25% was detected in neuronal dendritic spines and remaining 25% was observed in astrocytes (Milner et al., 2001). ERβ immunoreactivity was reported in the perikarya and proximal dendrites of hippocampal pyramidal and granule cells, as well as in non-principal cells of CA3 region of HIP. At the subcellular level, ERβ was affiliated with endomembranes, mitochondria and plasma membranes. Its immunoreactivity was also observed in both, dendritic shafts and spines, preterminal axons and axon terminals associated with synaptic vesicles (Milner et al., 2005). Similar to the AR, ERs are intracellular receptors influencing gene expression via hormone response elements occurring within hours or days (Bagamasbad & Denver, 2011). In addition, rapid, non-classical mechanism of E action dependent on membrane ERs has been previously described (Soltysik & Czekaj, 2013). It has been shown that binding of caveolin-1 is fundamental step of ERα and ERβ joining of cell membrane and a so-called palmitoylation of the receptor is necessary for ERs localization to caveolaes (cell membrane invaginations) (Acconcia et al., 2005; Pedram et al., 2007; Schlegel et al., 1999).

ERs can be localized at the plasma membranes in association with receptor tyrosine kinases (EGFR, IGF), G-proteins, striatin or Src tyrosine kinases (Levin, 2005; Xu et al., 2017). There are currently known two isoforms of intracellular ER receptors: ERα and ERβ. Both act as homodimers (β/β, α/α), but can also create a heterodimer (α/β) (Li et al., 2004). Different combinations of these units can be activated by various ligands and, in turn, exert tissue-specific actions mediated by binding to different transcriptional coactivators and corepressor proteins, leading to either agonist or antagonist action (selective ER modulators) (Kansra et al., 2005). Various estrogens have different affinities to these ERs: 17β-estradiol (E_2_) has equal affinity to both, whereas estrone preferentially binds to ERα and estriol to ERβ (Zhu et al., 2006).

ERα is crucial for the physiological development and functions of many organs and systems, such as reproductive, central nervous, cardiovascular and skeletal systems (Menazza et al., 2017; Ruiz et al., 2020; Vidal et al., 1999). The stress response induced by corticosterone can be modulated by the activation of ERα in the brain, ovary and uterus (Niranjan & Srivastava, 2019). ERα signaling has a pivotal role in the regulation of metabolic processes as it enhances insulin resistance, energy metabolism and mitochondrial function in an ovariectomized mouse model of metabolic syndrome (Hamilton et al., 2016). Obesity, specifically fat accumulation, can be prevented through the activation of ERα, which leads to enhancement of the energy expenditure (Arao et al., 2018). Moreover, the importance of ERα in regulation of obesity has been shown in female ERα knockout mice displaying worsened insulin resistance and higher adiposity, and in turn, enlarged size of early atherosclerotic lesions. Thus, ERα signaling could serve as a control point of atherosclerosis in females, because it can promote HDL function, liver cholesterol uptake and whole-body cholesterol removal. Moreover, the signaling cascade of ERα can protect females against the development of atherosclerosis (Zhu et al., 2018). In a mouse model of obesity, the features of metabolic syndrome, such as adiposity, plasma triglycerides, and oxidative stress, were reduced following administration of the grape seed extract enriched in the flavan-3-ols procyanidin dimers (the most effective red wine polyphenol on the endothelium) partially via ERα (Leonetti et al., 2018). Regarding the learning and memory, administration of the ERα selective agonist PPT (propyl pyrazole triol) to ovariectomized mice result in failure to learn the socially acquired preference of food. This outcome suggests, that ERα signaling could be impairing the memory for the socially acquired food preference (Clipperton et al., 2008).

As well as AR, ERα can activate the non-classical, rapid signaling cascade through its association with the cell plasma membrane. This signaling, together with the caveolin-binding protein striatin, activates MAPK, phosphatidylinositol 3-kinase and Akt kinase in the vascular endothelial cells leading to higher activity of endothelial NO synthase (Lu et al., 2004). These rapid actions can also increase glycerol release, lipolysis and induce beiging of adipocytes (Santos et al., 2018). In addition, reduction of cardiac ischemia reperfusion injury in endothelial cells of ovariectomized mice was observed following an estrogen-dendrimer conjugate treatment, which selectively activates non-classical ERα, in comparison to control mice (Menazza et al., 2017). Moreover, membrane-associated ERα signaling has an important role in bone growth (Iravani et al., 2017) and in vibration-induced effects of bone fracture healing (Santos et al., 2018). ERs rapidly affect neural plasticity (within 1 h), in a rapid learning experiment, the ovariectomized mice were tested within 40 min after ERα agonist PPT or ERβ agonist DPN (diarylpropionitrile) administration for social recognition, object recognition, or object placement learning. Results from this experiment showed that PPT administration improved social recognition, promoted object recognition and placement, and increased dendritic spine density in the stratum radiatum and lacunosum- molecular, which suggest that rapid E mediated learning enhancements may predominantly be mediated via ERα (Phan et al., 2011).

ERβ is important for migration of neurons and glial cells, or neural differentiation of embryonic stem cells, especially for differentiation of midbrain neurons (Varshney et al., 2017). Furthermore, dysregulation of ERβ signaling could have a negative effect on development of neurological disorders, such as dyslexia, through DNA de-methylation actions (Varshney & Nalvarte, 2017). ERβ also mediates calcium-induced mitochondrial permeability transition pore caused by ischemic brain injury through cyclophilin D and ATPase interaction (Burstein et al., 2018). In cancer research, ERβ seems to be a potential therapeutic target for colorectal or breast cancer, because its activation represses oncogenesis and metastasis (Austin et al., 2018; Williams et al., 2016). ERβ increases protein p53 signaling, leading to DNA repair (Weige et al., 2012), apoptosis and reduced proliferation (Hsu et al., 2006). In addition, the activation of ERβ signaling supports innate immunity resulting in the suppression of the cancer metastasis in lungs (Zhao et al., 2018). Concerning the other roles of this receptor, ERβ is involved in fat metabolism, where its activation induces fat mass redistribution and regulates hepatic triglyceride composition, which leads to tissue-specific and sex-dependent response to metabolic adaptation to overfeeding (González-Granillo et al., 2020). With reference to social learning, the ovariectomized mice treated with the ERβ selective agonist WAY-200070 (benzoxazole) showed a 2-fold prolonged preference for food eaten by their demonstrator. The results after the ERβ selective agonist treatment suggest that the enhancing effects on social learning may be due to the action of ERβ on submissive behavior (Clipperton et al., 2008). Another selective ERβ agonist - ISP358–2 (A-C estrogen), seems to be a potent candidate for enhancing memory consolidation in postmenopausal women (Hanson et al., 2018).

**Fig. 2.**
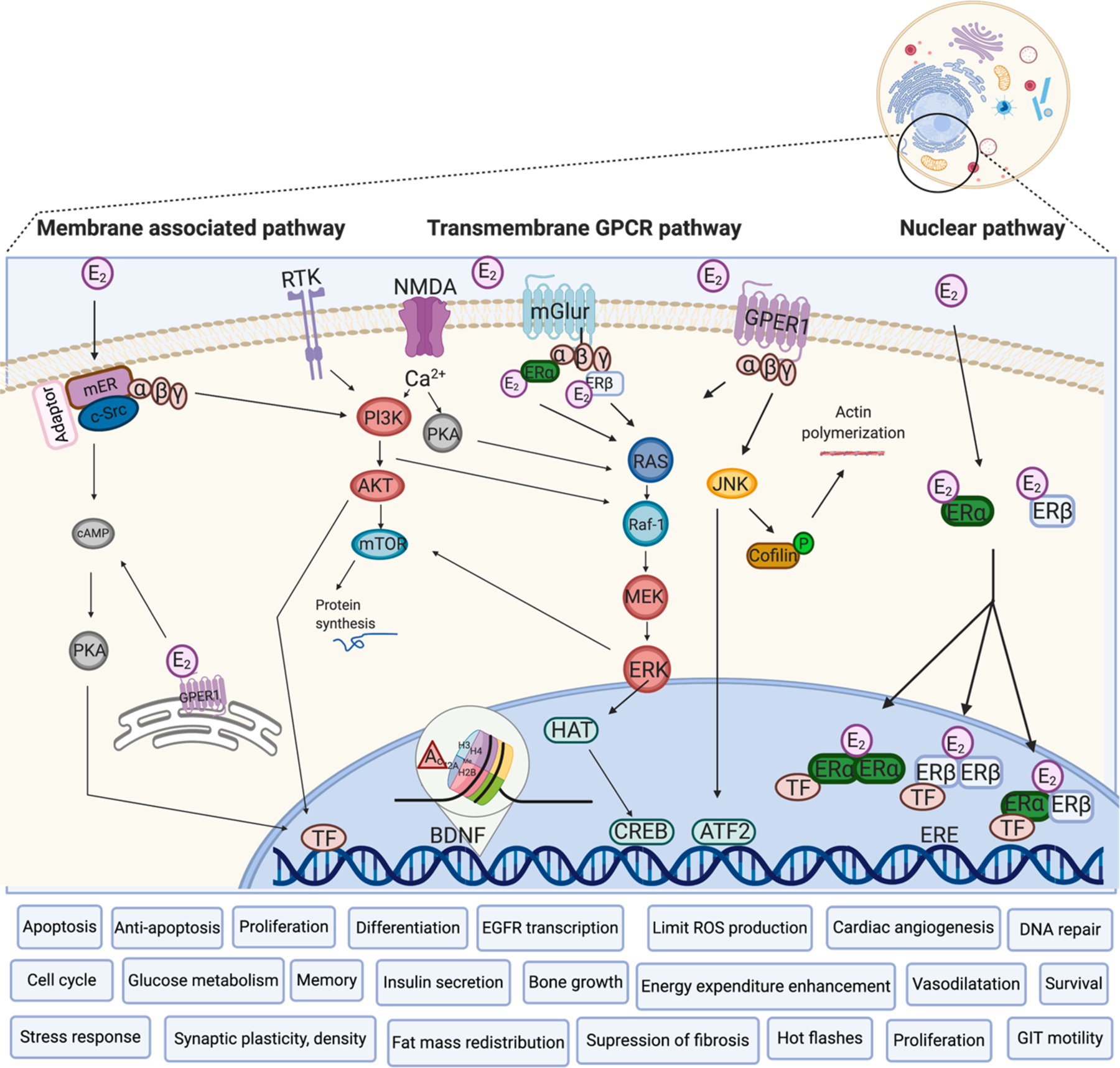
Intracellular, membrane-associated and transmembrane GPCR receptors of estrogens. E_2_ – Estradiol, ERα – Estrogen Receptor Alpha, ERβ– Estrogen Receptor Beta, mER – membrane-associated Estrogen Receptor, GPER1 – G-Protein Coupled Estrogen Receptor 1, mGLUR – metabotropic Glutamate Receptor, NMDA – N-Methyl-D-Aspartate receptor, RTK – Receptor Tyrosine Kinase, ERE – Estrogen Responsive Element, c-Src – protooncogene tyrosine-protein kinase Src, MEK – Mitogen-activated protein kinase kinase, AKT – protein kinase B, RAS – small GTPases, RAF-1 – proto-oncogene, serine/threonine kinase, CREB – cAMP Response Element-Binding protein, PI3K – PhospoInositide 3-Kinase, BDNF – Brain-Derivated Neurotrophic Factor, HAT – Histon AcetylTansferase, Ac – Acetyl group, mTOR – mammalian Target of Rapamycin, cAMP – cyclic Adenosine MonoPhosphate, PKA – Protein Kinase A, JNK – c-Jun N-terminal Kinases, ATF – Activating Transcription Factors, ERK – Extracellular signal-Regulated Kinases, TF – Transcription Factor, Ca2+ – Calcium (Adapted from (Carrier et al., 2015; Frick, 2015; Kim et al., 2019a; Pedram et al., 2008; Tu & Jufri, 2013) and created with BioRender.com).

Concerning the non-classical effects of ERβ, in cardiovascular system, especially in cardiomyocytes, ERβ activation stimulates PI3 kinase which increase the modulatory calcineurin-interacting protein 1 gene and protein expression, which subsequently inhibits calcineurin activity increased by angiotensin II and prevent the hypertrophy (Pedram et al., 2008). The classical effects of ERβ are also involved, where the transcription of the natriuretic peptide genes (ANP, BNP) are stimulated, whose as a proteins inhibit hypertrophy (activated by angiotensin II) via ERK signaling (Pedram et al., 2008). ERβ signaling also reverts pre-existing severe heart failure by stimulation of cardiac angiogenesis, suppression of fibrosis, and restoration of hemodynamic parameters. It has also been reported that ERβ membrane-associated receptors can mediate the reward circuitry in the brain and affect motivated behavior in females (Iorga et al., 2018). In older women, the period of the menopause is known not only for decline in cognitive function and impaired memory, but also for so called “hot flashes” that can be modified through ERβ activation. Administration of the selective agonist of ERβ - EGX358, reduced the senktide-mediated increase in tail skin temperature and enhanced memory in the object recognition and object placement tasks in ovariectomized mice. Thus, this ERβ agonist seems to be a promising in research of drugs for reducing menopause-related hot flashes or memory dysfunction (Fleischer et al., 2020). The possible signaling pathways of intracellular and membrane-associated ERs are summarized in Fig. 2. Besides intracellular or membrane associated receptors, E_2_ as well as T, binds to GPCR receptors.

#### 2.1.3. GPCR receptors for androgens and estrogens

The discovery of SSHs actions through GPCR transmembrane receptors is relatively new. A transmembrane AR called AR2 was discovered in the brain and gonads in 1999 (Sperry & Thomas, 1999). In 2014, was AR2 identified in Atlantic croaker ovaries as Zinc transporter protein 9/Zrt- and Irt-like protein 9 (ZIP9) 7-transmembrane G-protein coupled receptor (Berg et al., 2014; Thomas et al., 2014). G-protein coupled estrogen receptor (GPER1) was discovered in 1997 in ER-positive breast carcinoma cell lines and was initially called G protein-coupled receptor 30 (Carmeci et al., 1997). In 2005, it was established that this 7-transmembrane G-protein coupled receptor has a high affinity for E_2_ (Revankar et al., 2005), and thus, it was renamed GPER1.

##### 2.1.3.1. ZIP9xxx.

ZIP9, also known as zinc transporter protein, is a part of the 14-member ZIP family and is located in plasma and mitochondrial membrane, nucleus and endoplasmic reticulum (Thomas et al., 2014). ZIP proteins belong to the solute carrier family that manage membrane transport of zinc and regulates its cytoplasm concentration. Part of the zinc transporters regulate the efflux of zinc out of the cell and into vesicles (solute carrier 30) and other zinc transporters control the influx of zinc from outside the cell and from vesicles (solute carrier 39A or ZIP 1–14) (Berg et al., 2014; Thomas et al., 2014). The ZIP9 gene is mostly expressed in gonadal tissue and the brain (Berg et al., 2014). ZIP9 transmembrane receptor contains a ligand-binding groove for T which binds with high affinity to this receptor. Interestingly, there is a suggestion that monomeric ZIP9 might not represent the physiological state of its action in cells and that receptor needs to dimerize for T agonistic action (Kalyvianaki et al., 2019).

Regarding the signaling pathways, ZIP9 induces the phosphorylation of AKT and ERK through inhibition of protein tyrosine phosphatase, which leads to activation of B-cell receptor signaling in DT-40 cells (Taniguchi et al., 2013). ZIP9 can also induce the phosphorylation of ERK 1/2 and transcription factors – cAMP response element-binding protein (CREB) and activating transcription factor 1 (ATF1) – in Sertoli cells leading to claudin expression and tight junction formation. This ZIP9 cascade may be crucial for male fertility (Bulldan et al., 2016) because the Sertoli cells have a central role in spermatogenesis (Griswold, 1998). Upstream regulation of ZIP9 is controlled by inhibition of Notch signaling, which increases the expression of membrane ZIP9 and intracellular AR receptors as well as androgen-regulated claudin-5, claudin-11 and cAMP in mouse Sertoli cells (Kaminska et al., 2020). Epigenetics also plays a role in the regulatory effects of ZIP9, e.g. in skin, radiation-induced DNA-methylation leads to skin fibrosis via the ZIP9 and TGFβ signaling pathway (Qiu et al., 2020). Interestingly, before ZIP9 identification, it was shown that androgens can modulate zinc homeostasis in the mouse brain. Although the temporal and spatial zinc homeostasis in the brain is modulated by SSHs, the mechanisms and potential involvement of ZIP9 are still unknown (Beltramini et al., 2004).

##### 2.1.3.2. GPER1xxx.

GPER1 is mainly localized in the endoplasmic reticulum (Revankar et al., 2005), but is also located in the plasma membrane (Filardo et al., 2007). In brain, expression of GPER1 was detected in the cortex, HYP (paraventricular and supraoptic nuclei), HIP, specific nuclei of midbrain (the pontine nuclei, locus coeruleus), trigeminal nuclei, CER (Purkinje layer) and pituitary gland (anterior, intermediate, and neural lobes). The expression was detected also in other body systems such as cardiovascular (Almey et al., 2015; Hazell et al., 2009; Meyer et al., 2012), both female and male reproductive systems (Plante et al., 2012; Sandner et al., 2014), excretory system (Hazell et al., 2009) and gastrointestinal tract (Liu et al., 2019b).

The impact of GPER1 signaling on the brain development and functions is not clear, but GPER1 is highly expressed in the nervous system, and its activation shows beneficial, cell specific effects in various brain disorders (Alzheimer’s, Parkinson’s disease) (Roque et al., 2019; Sheppard et al., 2018). It has been found that GnRH secretion in the HPG axis is modulated by GPER1 (Chimento et al., 2014). The signaling cascade through activation of GPER1 includes various non-classical actions that seem to be tissue specific. The stimulation of GPER1 activates Ca^2+^ release, ERK1/2, PI3K action and stimulation of epidermal growth factor receptor (EGFR) transcription in breast cancer cell lines (Filardo et al., 2000). In the dorsal HIP of female mice, GPER1 does not activate ERK1/2, but rather signals through c-Jun N-terminal kinase (JNK) phosphorylation instead (Filardo et al., 2000). GPER1 stimulation in the hippocampus can lead to better performance in spatial working memory tasks in ovariectomized rats (Hammond et al., 2009) and can improve object and spatial memory consolidation in ovariectomized mice (Kim et al., 2016c). However, unlike ERα and ERβ, GPER does not activate ERK in the dorsal HIP nor is dorsal HIP ERK activation necessary for GPER to influence object recognition and spatial memory consolidation in ovariectomized mice (Kim et al., 2016a). Regarding the epilepsy and HIP, the reduction of seizures’ severity has been observed after GPER1 activation (Zuo et al., 2020). Moreover, higher concentrations of GPER1 in the dorsal prefrontal cortex of monkeys was associated with greater dendritic spine synapse density in this area, suggesting an important role for GPER1 in synaptic plasticity (Crimins et al., 2016).

E_2_ binds GPER1 with high affinity and this activation leads to ERK phosphorylation, PI3K stimulation, intracellular Ca^2+^ increase, and cAMP production in the MCF-7 breast cancer cell line, which occurs via trans-activation of the epidermal growth factor receptor and results in proliferation (Filardo et al., 2000). Although, GPER1 mediates proliferation in the human breast epithelial cells in normal and malignant breasts, GPER1 knockout mice do not show any overt mammary phenotype similar to ERβ knockout mice. It means that both GPER1 and ERβ operate breast tissue proliferation but only ERα signaling is crucial for breast development (Scaling et al., 2014). In males, GPER1 has a critical role in spermatogenesis, where it controls proliferation and apoptosis (Chimento et al., 2014).

GPER1 signaling seems to have plenty of more functions in different body systems. For example, it preserved degeneration of retinal ganglion cells and acute ocular hypertension through the PI3K/AKT pathway (Jiang et al., 2019). Through the AKT/mTOR/GLUT pathway, GPER1 manages glucose metabolism and insulin secretion in β-cells of rats (Bian et al., 2019b). In myenteric neurons of the gastrointestinal tract the GPER1, as well as ERs, plays a role in motility (Liu et al., 2019a). In the endothelium of blood vessels, GPER1 activation leads to vasodilatation (Meyer et al., 2012). Furthermore, GPER1 seems to be a potential therapeutic target for females after menopause suffering from salt-sensitive hypertension (Gohar et al., 2020). In cardiac cells (cardiomyocytes, cardiac fibroblasts, mast cells), GPER1 signaling inhibits the gene expression of components (cyclin B1, CDK1) involved in proliferation of cardiac fibroblasts and mast cells, and prevents hypertrophic remodeling (Deschamps & Murphy, 2009; Ogola et al., 2019; Wang et al., 2012). Cardiovascular and kidney protection via GPER1 has been studied by the examination of angiotensin II-induced hypertension and oxidative stress in GPER1 knockout mice. Estrogen signaling through GPER1 suppresses the transcription of NADPH oxidase 4 by increasing cAMP, thereby limiting the production of reactive oxygen species which avoids stiffening of the arteries (Ogola et al., 2019).

Effects of GPER1 signaling in the brain and other organs are summarized in Table 1. In the next part of the review, the effects of T and E_2_ signaling in selected brain regions (HIP, HYP, FC, and CER) will be discussed.

## 3. The effect of T and E_2_ on brain structures

During fetal development, the brain is already being influenced by sex hormones (T, E, Progesterone) and neurons throughout the entire nervous system already have receptors for these sex hormones (Karaismailoğlu & Erdem, 2013; Miranda & Sousa, 2018; Swaab & Garcia-Falgueras, 2009). Prenatal and early postnatal SSHs exposure organizes the brain in a male-typical or female-typical pattern. These sex-typical differences in brain structures are partially results of the organizational effects of SSHs that can have long-term influence on dendritic spine remodeling, myelination, neuronal pruning, apoptosis, and/or epigenetic changes later in adulthood. SSHs have also activational effects on aforementioned changes in the brain (dendritic spine remodeling, myelination, etc.) later in life and those are dependent on hormone concentration in adulthood. The behavioral outcomes, such as copulation, spatial abilities or memory, are the consequences of both organizational and activational effects of SSHs (Vigil et al., 2016). The other important factors that regulate the sex-specific action of T and E_2_ are the enzymes converting T to E_2_ (aromatase) or DHT (5α-reductase). The dissimilarities in concentrations of androgens, estrogens, aromatase, and 5α-reductase, as well as in expression of SSH receptors (AR, ERs, ZIP9, GPER1) in specific areas of the brain contribute to behavioral heterogeneity between males and females (Colciago et al., 2005; Roselli et al., 1985). The role of androgens and estrogens in selected brain regions related to behavior, including the HIP, HYP, FC, and CER, are summarized in Table 2.

### 3.1. T and E_2_ in hippocampus

Androgens influence the structural development of the HIP by increasing and maintaining spine synaptic density in both males and females (Leranth et al., 2004, 2003; MacLusky et al., 2006). In male and female rats, testosterone proprionate rescues gonadectomy-induced reductions in CA1 spine synaptic density in a manner that partially depends on afferent subcortical input from fimbria-fornix, however, some of the effects are still present after fimbria-fornix transection (Kovacs et al., 2003). Moreover, T, as well as E_2_, increases cell density by stimulating neuronal cell proliferation in the HIP (Smeeth et al., 2020). As well as in early development and in puberty, neurogenesis provoked by T (or DHT) can occur during adulthood in both sexes (Okamoto et al., 2012; Spritzer & Roy, 2020). However, there are sex differences in early development of the HIP, where cell proliferation during the first postnatal week is approximately 2-times higher in male compared to female rodents (Bowers et al., 2010). Moreover, neonatal male rats have a significantly higher number of cells in the HIP than female rats (Zhang et al., 2008). T can also increase synaptic density in the dentate gyrus and promote neurogenesis in HIP of male, but not female, mice (Fattoretti et al., 2019).

In males, synaptic plasticity in CA1 pyramidal neurons is affected by androgens through AR (Islam et al., 2020). Furthermore, the activation of AR-dependent signaling in the dentate gyrus increases survival of adult-born neurons in male rats (Hamson et al., 2013). In another experiment, the replacement of T in gonadectomized male rats alleviated impaired memory caused by a reduction of AR-immunoreactive neurons in the male HIP (Moghadami et al., 2016). In hippocampal primary neuron cultures, T treatment rapidly increased spine density through non-classical cascades such as increased expression of phosphorylated ERK1/2 and CREB, but not the p38 and JNK (Guo et al., 2020). In addition, T-stimulated synaptic plasticity in the HIP could be mediated through brain-derived neurotrophic factor (BDNF) (Atwi et al., 2014). Concerning clinical studies, in middle-aged males with higher cortisol concentrations, plasma T concentrations were positively associated with hippocampal volume and memory performance (episodic memory), suggesting that T also exerts neuroprotective effects in men (Panizzon et al., 2018). Based on these studies, T seems to be important for many hippocampal functions and deserves attention as a regulator of synaptic plasticity and memory.

Regarding the actions of E_2_ in the HIP, dorsal hippocampal administration of E_2_ rapidly enhances object recognition and spatial memory consolidation through many mechanisms, including increased acetylation of histone 3 at several *Bdnf* promoters and lower expression of histone deacetylase proteins (Fortress et al., 2014; Tuscher et al., 2018). The activation of GPER1 by direct infusion of GPER1 agonist G1 into the dorsal HIP facilitates object recognition memory and hippocampal-dependent spatial memory in ovariectomized female mice via phosphorylation of JNK, which leads to cofilin-mediated actin polymerization and spinogenesis (Kim et al., 2019a, 2016a). The important role of ERα during the early developmental period, as in development of reproductive tissue, but also a non-reproductive role in developing brain, has been emphasized (Bondesson et al., 2015). The hippocampal ERα signaling activation stimulates glutamatergic synapse formation during development (Jelks et al., 2007). Additionally, the expression of ERα (Solum & Handa, 2001) and the colocalization of ERα and BDNF in pyramidal cells of CA3 and CA1 subregions of HIP occurs. This interaction between ERα and BDNF could modify the physiology of HIP during development (Solum & Handa, 2002). Concerning ERα-mediated gene transcription in the nucleus, ERβ seems to be a negative regulator of this action (Bean et al., 2014). On the other hand, studies of long-term treatment with an ERβ agonist (diarylpropionitrile) have shown that ERβ signaling contributes to regulation of neurogenesis, neuro-modulation and neuroprotection in the hippocampal formation of ovariectomized middle-aged rats (Sárvári et al., 2016).

### 3.2. T and E_2_ in hypothalamus

The most important role of the HYP is linking the nervous system to the endocrine system through the anterior (adenohypophysis) and posterior (neurohypophysis) parts of the pituitary gland (hypothalamo-pituitary axis); therefore, it supports body homeostasis by regulation of endocrine system and autonomic behavior (Namwanje & Brown, 2016; Vadakkadath Meethal & Atwood, 2005). Neurons in HYP are also necessary for various types of learning and memory (Burdakov & Peleg-Raibstein, 2020). Moreover, the HYP has an important role in regulation of metabolism, energy expenditure and gastrointestinal tract via the brain-gut-microbiota axis. There are suggestions that interaction between mental stress and gut microbiota may affect the development of hypothalamic–pituitaryadrenal axis itself (Frankiensztajn et al., 2020).

Androgens can also stimulate morphological maturation of hypothalamic aromatase-immunoreactive neurons in mouse embryos and, therefore, may influence the synaptic plasticity and connectivity in hypothalamic aromatase-system (Beyer & Hutchison, 1997). It has been shown that aromatase activity in male HYP neurons is similar to that of females, but that males have a higher percentage of neurons expressing aromatase (Beyer et al., 1994). In rats, T, but also the nonsteroidal antiandrogen (flutamide), administered both prenatally and postnatally affected the development of sexually dimorphic nuclei in HYP by increasing cell volume and length (Lund et al., 2000). The T-mediated regulation of the expression of AR, ERs and aromatase in the HYP is age dependent. In adolescence, the corticosterone release is regulated mostly by conversion of T to E_2,_ while in adulthood greater conversion of T to DHT occurs in male rats (Green et al., 2019).

Sex chromosomes (especially XY) manage early development of HYP neurons. This management involves the sex specific neuritogenesis and regulation of the process of neuritogenic factor neurogenin 3 expression stimulated by ERα signaling in the HYP (Cisternas et al., 2020). It was also shown that neonatal E administration can modify the synapse formation of the hypothalamic arcuate nucleus (Arai & Matsumoto, 1978).

E_2_ promotes many actions in the HYP. For example, it increases the expression of glial cell neurotrophic factor in hypothalamic neurons, but not in astrocytes, through non-classical E action (calcium, cAMP/PKA) (Ivanova et al., 2002). E_2_ also stimulates caspase-dependent cell death in the developing HYP and regulates tyrosine hydroxylase-labelled dopaminergic neurons in the anteroventral periventricular nucleus of the HYP, in which intracellular hormone receptors are abundant (Waters & Simerly, 2009). Moreover, neonatal administration of E_2_ can elevate the number of axodendritic synapses in the hypothalamic arcuate nucleus (Matsumoto & Arai, 1976). In addition, E_2_ stimulates axogenesis in male HYP via ERK1/2 and ryanodine receptors-dependent intracellular calcium rise (Cabrera Zapata et al., 2019).

### 3.3. T and E_2_ in frontal cortex

Androgens seem to be critical modulators of executive functions in the mesocorticolimbic area, including the prefrontal cortex (PFC) (Tobiansky et al., 2018a). Early exposure (postnatal day 10) to T leads to hyperactivity, higher impulsivity and attention deficit behavior in individuals who already have the genetic predisposition for these behaviors (King et al., 2000). During puberty, there is a shift of emotional control from the pulvinar nucleus of the thalamus and amygdala to the anterior PFC caused by T (Tyborowska et al., 2016). T can also affect emotions in the PFC of psychopathic offenders, where patients, especially those with high endogenous T concentration, show less emotional control-related anterior PFC activity and anterior PFC-amygdala coupling during the tests of emotional actions (Volman et al., 2016). High concentrations of T have been associated with lower cortical density in the left hemisphere, especially in the PFC of prepubertal boys, and these effects are associated with higher aggression and lower executive function. In addition, Nguyen et al. (2016) have found that T-specific modulation of the covariance between the amygdala and medial PFC could influence and predict aggressive behavior from childhood to adulthood (Nguyen et al., 2016). Moreover, elevated concentrations of T are associated with increased risk-taking in both genders, independent of age. More risk-taking is associated with a smaller orbitofrontal cortex in males and larger orbitofrontal cortex in females (Peper et al., 2013).

Unlike higher concentration of T, DHEA is associated with a prepubertal increase in neuron density in various cortical regions, which can positively facilitate cortical functions such as attention and working memory (Nguyen, 2018). Male anabolic–androgenic steroid users have thinner PFC areas involved in inhibitory control and emotional regulation (Hauger et al., 2019). Furthermore, long-term anabolic–androgenic steroid use results in executive dysfunction, including ADHD symptoms or psychological distress (Hauger et al., 2019), and also anxiety, depression, aggressive or antisocial behavior (Kashkin & Kleber, 1989).

The early expression of ERs and GPER1 was detected in the dorsal FC, ventral FC and the HIP during the developmental period spanning embryonic to late prenatal (Denley et al., 2018). Moreover, prenatal E_2_ modulates the development of catecholamine activity during neonatal period in male FC (Stewart & Rajabi, 1994). Puberty presents another sensitive period in brain development. The organizational effects of E_2_ and progesterone promote the maturation and increase of inhibitory neurotransmission in FC in pubertal females (males not studied here) (Piekarski et al., 2017). Moreover, Chung et al. (2019) have found age-dependent association between E_2_ concentrations and emotional activity in dorsolateral PFC, where adolescents with higher E_2_ concentrations showed positive reconsideration of negative emotions (Chung et al., 2019). In adults, the medial PFC together with the dorsal HIP is responsible and critical for object memory and spatial memory consolidation mediated by E_2_ (Tuscher et al., 2019).

The sex dependent impact on cognitive and synaptic function may be attributed to the fact that females PFC contain higher concentrations of aromatase than males. Higher E concentration in the PFC protects females against harmful effects of repeated stress in comparison to males (Yuen et al., 2016). E_2_ concentration in FC could also affect executive function, such as working memory, organization, planning and sustained attention (Hampson, 2018; Shanmugan & Epperson, 2014). Moreover, executive functioning in the PFC depends on E_2_ concentrations, together not only with molecules such as dopamine, norepinephrine, serotonin, and acetylcholine, but also with genetic conditions, stress, early life experiences and lifestyle choices (Shanmugan & Epperson, 2014).

### 3.4. T and E_2_ in cerebellum

In the CER, Purkinje cells have been identified as the main site of neurosteroid synthesis in the cerebellum of most vertebrates (Tsutsui, 2012). Moreover, the expression of AR in Purkinje neurons has been found, and the AR concentration can be modified by systemic T, through alteration of AR in specific regions, and by sexual behavior-induced reductions of AR in the posterior vermis (Perez-Pouchoulen et al., 2016b). The density of AR is also altered in the posterior CER after prenatal administration of valproic acid, which influenced the development of CER in both males and females age-dependently (Perez-Pouchoulen et al., 2016a).

It has been also reported that the volume of the CER negatively correlates with neurotic personality traits in adolescents and young adults, where higher endogenous T concentration was related to thicker CER gray matter volume and lower neuroticism score (Schutter et al., 2017). T seems to have protective effects on cerebellar neurons. For example, T treatment can reverse the age-related increase in glial fibrillary acidic protein (GFAP) in the male CER (Day et al., 1998). Moreover, T displays protective effect on CER granule cells against the oxidative stress through the AR (Ahlbom et al., 2001). Regarding neurodegenerative disorders, T seems to be an appropriate biomarker of spinocerebellar ataxia type 2 progression, where 35% reduction of T concentration in male patients was detected (Almaguer-Mederos et al., 2020).

The most potent estrogen, E_2_, has a higher impact on the developing brain (dendritic spinogenesis, synapse formation, cell proliferation and apoptosis, neuronal differentiation) in comparison to adulthood (McCarthy, 2008). At the second hour of life, concentrations of E_2_ are higher in the FC, HYP, and preoptic area (but not in the CER, HIP and brainstem) of male rats, and highest in the female rat HIP in comparison to other brain regions of female rats. During the first PND, the concentrations of E_2_ decreased in the majority of brain regions and the only sex difference remained in its hypothalamic concentrations (Amateau et al., 2004).

ERβ signaling is involved in regulation of cell growth during cell differentiation in the developing CER of male and female rat pups (Jakab et al., 2001). Moreover, early childhood inflammation in the CER supports synthesis of E_2_ during sensitive periods (windows), which begin at about 1 year of age (Wright et al., 2019). During this sensitive period, the synthesis of E_2_ plays a crucial role in cerebellar Purkinje cells. This process can be disrupted by inflammation with long-term consequences, but has been observed only in males (Hoffman et al., 2016). Furthermore, endogenous E_2_ has been shown to affect microglial phagocytosis during the sensitive window of postnatal development of the CER (Perez-Pouchoulen et al., 2019). E_2_ also regulates the neurotransmission of parallel fibers to Purkinje cells in the CER (Hedges et al., 2018). E_2_ is expressed in cerebellar granule cells (Belcher, 1999) and has a trophic effects on these cells in both, males and females (Montelli et al., 2017). In the experiment with chicken cerebellar granule neurons, E_2_ can protect these granule cells against glutamate-induced toxicity both, acutely and long-term, depending on E_2_ concentrations (Sørvik & Paulsen, 2017). The E_2_ can also modulate motor memory formation in the adult male CER (Dieni et al., 2018). *De novo* synthesized E_2_ modulates CER functions through cerebellar neurotransmission and CER-dependent learning (Dieni et al., 2020). Furthermore, the improvement of cerebellar memory by E_2_ is mediated through ERβ (Andreescu et al., 2007).

## 4. Conclusions

SSHs are crucial for the proper development and function of the body in both, males and females. The research regarding the effect of SSHs on different organs and body systems, especially the brain, is moving forward very quickly, and it is important to stay abreast of the latest developments. The present review summarizes the latest information on the effects of SSHs (e.g. T and E_2_) via their classical or non-classical pathways at functional, cellular, and tissue levels, with the main focus on brain regions involved in cognition, including the HIP, HYP, FC and CER. The role of SSHs in modulation of behavior in both humans and laboratory animals is described. SSHs are involved in regulation of many body systems such as reproductive, immune, muscular, cardiovascular, skeletal and neural. SSHs affect these systems differently via different receptors. Although the actions of intracellular AR are central for example for bone healing, glucose and fat metabolism and β-amyloid plaque reduction, the role of membrane-associated AR is pivotal in process of neuronal apoptosis, cell survival and cell proliferation. ERα plays an important role in energy metabolism, insulin resistance, fat accumulation or atherosclerosis. On the other hand, the fast, non-classical actions of ERα result in endothelial NO activation, lipolysis, bone growth and beiging of adipocytes. Intracellular ERβ is involved in neural differentiation and in oncogenesis and metastasis suppression, whereas non-classical ERβ signaling can reverse pre-existing heart failure or inhibit hypertrophy of cardiomyocytes. Concerning transmembrane GPCR receptors, ZIP9 is crucial for male fertility, spermatogenesis, or apoptosis, whereas GPER1 is responsible for proper functioning of gonads in both males and females.

Regarding the effects of T or E_2_ on HIP, HYP, FC and CER, activation of the appropriate receptor triggers changes in both brain structures and behavior. For example, T increases synaptic spine density and neurogenesis in HIP, and increases synaptic plasticity and connectivity. In addition, it modulates the morphological maturation of HYP, cortical density in the FC and increases the gray matter volume in the CER. In addition, T improves working and spatial or episodic memory (HIP), increases male sexual or aggressive behavior (HYP), regulates executive functions and control of emotions (FC), and decreases neuroticism and regulation of male sexual behavior (CER). E_2_ increases glutaminergic synapse formation and neurogenesis in HIP, modulates synapse formation, increases axodendritic synapses and axogenesis in HYP, and regulates neurotransmission in the FC and cell growth in the CER. E_2_ also improves object recognition and spatial memory consolidation (HIP, FC), controls aggressive behavior and anorectic actions in females (HYP), and regulates modulates motor memory formation (CER).

Both classical and non-classical actions of T and E_2_ in the brain confirm the importance of these SSHs in regulation of structural changes in the brain with an impact on behavior and cognitive function. Plenty of studies have been published describing the molecular signaling pathways of SSHs receptors and their effects on the brain, body systems and behavior. The results of these studies have brought new insights into the neurobehavioral effects of T and E_2_. However, additional questions have arisen, such as the sex-, age- and tissue-specific role of rapid, non-classical mechanisms involving the GPCR ZIP9 and GPER1 receptors, mainly the brain. The answers to these questions will provide a more complete picture of how SSHs regulate the functional of neural and non-neural systems.

## Figures and Tables

**Table 1:** The classical and non-classical actions of SSHs through their receptors in the cytoplasm, those attached to membrane (AR, ERs) and through transmembrane GPCR receptors (ZIP9, GPER1).

H.	R.	Type	Signaling pathway	Effect on the brain	Effect on the other body systems or on the behavior	
**Testosterone, Dihydrotestosterone**	**AR**	**Intracellular**	Regulation of gene transcription in the nucleus (more than 3000 genes (Jin et al., 2013)): IGF receptor (Pandini et al., 2005), KLK3 (PSA), KLK2, and NKX3-1 (Prescott et al., 1998) DNA binding motif: AGAACA (Ikeda et al., 2015)	organizational and activational effect brain masculinization (Sato et al., 2004) GnRH expression regulation in HYP (Belsham et al., 1998; Belsham & Lovejoy, 2005) neurogenesis regulation, activation of growth factors, myogenesis (McCulloch et al., 2000) ↑ pyramidal neurons plasticity in HIP (Islam et al., 2020) protection against oxidative stress in CER (Ahlbom et al., 2001) decreased (β-amyloid plaques, decreased neuronal apoptosis (Pike et al., 2008)	protect memory consolidation (Koss & Frick, 2019) enhance inhibitory avoidance memory (Islam et al., 2021) anxiolytic and antidepressant effects in males (Chen et al., 2014)	regulation of hemopoietic system, glucose and fat metabolism (Lin et al., 2005) prostate epithelial homeostasis (Zhang et al., 2016) bone healing (Komrakova et al., 2020) muscle fast-twitch, hypertrophy (Davey et al., 2017; Morton et al., 2018) role in prostate and breast cancer (Debes & Tindall, 2002; Giovannelli et al., 2018)
		**Membrane-associated**	PI3K/AKT; PKA, PKC; Src/p85α/PI3K; Src/Shc/ERK; Src/p85α/p110/AKT/FOXO, Bad; ERK/MAPK; Src/p85α/MAPK/Elk1/CREB (Baron et al., 2004; Duarte et al., 2016; Kang et al., 2004; Kousteni et al., 2001; Lucas-Herald et al., 2017; Sukocheva et al., 2015)			↑ cell survival and proliferation (Liao et al., 2013)
	**ZIP9**	**GPCR**	AKT, ERK1/2; Bax, p53, JNK, caspase-3, cytochrome *c*; CREB, ATF1 cells (Bulldan et al., 2018, 2016b; Taniguchi et al., 2013; Thomas et al., 2014)	potential organizational and activational effect	activation of the B-cell receptor signaling in DT-40 cells (Taniguchi et al., 2013) male fertility (Bulldan et al., 2016) spermatogenesis (Griswold, 1998) ↑ skin fibrosis (Qiu et al., 2020)	
**Estrogens**	**ERα**	**Intracellular**	Regulation of gene transcription in the nucleus (more than 3000 genes (Welboren et al., 2007)): COX7RP, EBAG9 (Watanabe et al., 1998), Efp (Ikeda & Inoue, 2004), NRIP, Forkhead, AP-1, Oct and C/EBP motifs (Welboren et al., 2007) DNA binding motif : AGGTCA (Ikeda et al., 2015)	organizational and activational effect glutaminergic synapse formation (Jelks et al., 2007) memory formation, ↑ synapse plasticity, spine density, neurogenesis, (Baumler et al., 2019; Dominguez et al., 2018; Duarte-Guterman et al., 2015; Frick, 2015; Frick et al., 2015; Galea et al., 2018; Gibbs & Gabor, 2003; Kim et al., 2019b; Koss et al., 2018a; Sun et al., 2019) ↑ HIP-dependent memory consolidation (Kim et al., 2016b; Koss et al., 2018b; Krentzel et al., 2019) affect spatial memory, memory consolidation and cognitive functions (Duarte-Guterman et al., 2015; Frick, 2015; Frick et al., 2015; Galea et al., 2018; Koss et al., 2018a, 2018b) masculinization of HIP cholinergic system (Mitsushima et al., 2009a, 2009b) social recognition regulation (Pierman et al., 2008) brain protection: antioxidant defense and attenuation of apoptotic pathway (Baez-Jurado et al., 2019) neural differentiation 93	enhance learning via ERα (Phan et al., 2011) could impair the memory for socially acquired food preference via ERα (Clipperton et al., 2008) anxiolytic and antidepressant effects (Chhibber et al., 2017; Xu et al., 2016) control of aggressive behavior in female (Hashikawa et al., 2017) mediate the reward circuitry – affect motivated behavior in females via ERβ (Tonn Eisinger et al., 2018) enhance effect on social learning via ERβ (Clipperton et al., 2008) enhance memory consolidation via ERβ (Hanson et al., 2018)	modulation of stress response (Niranjan & Srivastava, 2019) ↑energy metabolism, mitochondrial function, insulin resistance (Hamilton et al., 2016) prevention of fat accumulation (Arao et al., 2018) atherosclerosis prevention – promote HDL function, liver cholesterol uptake, and whole-body cholesterol removal (Zhu et al., 2018) breast cancer development (Brisken & O’Malley, 2010)
		**Membrane-associated**	MAPK, AKT (171); PI3K, PKC, Ca2 influx, ERK; MD+M2/P53/Bcl2/Bax; P21/P27; GSK-3β/CyclinD1; Bad/Caspase9; NF-KB (Barone et al., 2010; Xu et al., 2017),			↑ endothelial NO synthase activity (Lu et al., 2004)↑ glycerol release, lipolysis, induce beiging of adipocytes (Santos et al., 2018)↓cardiac reperfusion injury in endothelial cells (Menazza et al., 2017)bone growth, fracture healing (Iravani et al., 2017; Santos et al., 2018)
	**ERβ**	**Intracellular**	Regulation of gene transcription in the nucleus (more than 3000 genes (Welboren et al., 2007)): COX7RP, EBAG9 (Watanabe et al., 1998), Efp (Ikeda & Inoue, 2004), NRIP, Forkhead, AP-1, Oct and C/EBP motifs (Welboren et al., 2007) DNA binding motif: AGGTCA (Ikeda et al., 2015)			↑ ANP, BNP, inhibit hypertrophy of cardiomyocytes (Pedram et al., 2008) affect the development of neurodevelopmental disorders via DNA-demethylation (Varshney & Nalvarte, 2017) stimulation of p53, oncogenesis and metastasis suppression (Austin et al., 2018; Williams et al., 2016) support innate immunity (Zhao et al., 2018) fat mass redistribution, regulate hepatic triglyceride composition (Gonzalez-Granillo et al., 2020)
		**Membrane-associated**	BDNF/TrkB (465); PI3K, PKC, Ca2 + influx, ERK; MDM2/P53/Bcl2/Bax; P21/P27; GSK-3β/CyclinD1; Bad/Casp9; NF-kB (Xu et al., 2017)			↑modulatory calcineurin-interactin protein 1, which inhibit calcineurin activity and hypertrophy of cardiomyocytes (Pedram et al., 2008) reverts pre-existing heart failure: ↑ angiogenesis, ↓fibrosis, hemodynamic parameters restoration (Iorga et al., 2018)
	**GPER1**	**GPCR**	Ca2+, cAMP, ERK1/2, PI3K; PI3K/AKT; AKT/mTOR/GLUT; (Bian et al., 2019a; Filardo et al., 2000) JNK, cofilin (Kim et al., 2019a)	potential organizational and activational effect spatio-temporal signalization in HIP (Evans, 2019) synaptic plasticity regulation and ↑ spine density (Crimins et al., 2016) ↓ epileptic seizures severity (Zuo et al., 2020) neuroprotective effects in various brain disorders (Roque et al., 2019) blood brain barrier protection (Maggioli et al., 2016) ↑ synaptic spine density (Frick, 2015; Kim et al., 2019b; Koss et al., 2018a, 2018b) modulation of GnRH secretion in HPG axis (Chimento et al., 2014)	anxiogenic and depressant effects in both males and females (Fındıklı et al., 2016; Kastenberger & Schwarzer, 2014; Krȩżel et al., 2001) ↑EGFR transcription, cell proliferation (Filardo et al., 2000) improve the object and spatial memory consolidation and working memory (Hammond et al., 2009; Kim et al., 2016c) preserved degeneration of retinal ganglion cells and acute ocular hypertension (Jiang et al., 2019) manages glucose metabolism and insulin secretion in β-cells (Bian et al., 2019b) ↑ gut motility and contraction (Liu et al., 2019a) vasodilatation (Meyer et al., 2012) prevent hypertrophic remodeling (Deschamps & Murphy, 2009; Ogola et al., 2019; Wang et al., 2012) ↓ reactive oxygen species, arteries stiffening prevention (Ogola et al., 2019)	

**Table 2:** The role of T and E_2_ in HIP, HYP, FC, and CER, brain regions related to cognition – experimental studies.

	Hippocampus	Hypothalamus	Frontal cortex	Cerebellum
**Testosterone – effect on brain structures**	↑ development synapse spine density (Leranth et al., 2004, 2003; MacLusky et al., 2006) ↑ neurogenesis (Okamoto et al., 2012; Smeeth et al., 2020; Spritzer & Roy, 2020) ↑ synaptic density in DG in aged males not females (Fattoretti et al., 2019) ↑ pyramidal neuron plasticity in CA1 in males (Islam et al., 2020) ↑ HIP volume in males (Panizzon et al., 2018)	development of sexually dimorphic nuclei (Lund et al., 2000) ↑ morphological maturation, synaptic plasticity and connectivity (Beyer & Hutchison, 1997) modulation of metabolic circuitry in neurons (Sheppard et al., 2011) ↑ somatostatin mRNA (Argente et al., 1990)	cortical density (Nguyen, 2018)	↑ gray matter volume (Schutter et al., 2017) prolonged ↓ of gray matter volume in males (Wierenga et al., 2018) protection of neurons, reverse increased concentration of GFAP (Day et al., 1998)
**Testosterone – effect on behavior**	↑ working and spatial memory regulation (Fattoretti et al., 2019; Hough et al., 2017; Koss & Frick, 2019) ↑ episodic memory in males (Panizzon et al., 2018)	↑ male sexual behavior (McGinnis et al., 1996) ↑male aggressive behavior (Bermond et al., 1982)	modulation of executive functions (Tobiansky et al., 2018a) emotion control after puberty (Tyborowska et al., 2016; Volman et al., 2016) ↑ aggressive behavior (Nguyen et al., 2016) ↑ risk taking (Peper et al., 2013) attention and working memory regulation (Nguyen, 2018)	regulation of the male sexual behavior (Perez-Pouchoulen et al., 2016b) ↓ neuroticism (Schutter et al., 2017)
**Estradiol–effect on brain structures**	↑ glutaminergic synapse formation (Jelks et al., 2007) ↑ neurogenesis (Smeeth et al., 2020) ↑ acetylation of BDNF and histone deacetylase proteins (Fortress et al., 2014; Tuscher et al., 2018) ↑ CA1 dendritic spine density (Frick, 2015; Kim et al., 2019b; Koss et al., 2018a, 2018b)	modification of the synapse formation of the arcuate nucleus (Arai & Matsumoto, 1978) ↑ glial cell line-derived neurotrophic factor concentration (Ivanova et al., 2002) induction of caspase dependent apoptosis and regulation of hydroxylase-labelled dopaminergic neurons (Waters & Simerly, 2009) ↑ axodendritic synapses and axogenesis (Cabrera Zapata et al., 2019; Matsumoto & Arai, 1976)	inhibitory neurotransmission regulation in pubertal females (Piekarski et al., 2017)	microglial phagocytosis modification (Perez-Pouchoulen et al., 2019) cell growth regulation during cell differentiation (Jakab et al., 2001) granule cells protection (Sørvik & Paulsen, 2017)
**Estradiol – effect on behavior**	regulation of object recognition, memory consolidation and spatial memory (Fortress et al., 2014; Frick & Kim, 2018; Frick et al., 2015; Kim et al., 2016a; Tuscher et al., 2016)	↑ anorectic actions (Shen et al., 2010) control of aggressive behavior in females (Hashikawa et al., 2017)	regulation of object and spatial memory consolidation (Carney, 2019) positive reconsideration of negative emotions (Chung et al., 2019)	motor memory formation modulation in adult males (Andreescu et al., 2007; Dieni et al., 2018) modulate motor learning (Dieni et al., 2020)

## References

[R1] Acconcia F, Ascenzi P, Bocedi A, Spisni E, Tomasi V, Trentalance A, Visca P, Marino M, 2005. Palmitoylation-dependent estrogen receptor alpha membrane localization: regulation by 17beta-estradiol. Mol. Biol. Cell 1. 10.1091/mbc.e04-07-0547. PMC539167 15496458

[R2] Ahlbom E, Prins GS, Ceccatelli S, 2001. Testosterone protects cerebellar granule cells from oxidative stress-induced cell death through a receptor mediated mechanism. Brain Res 2 10.1016/S0006-8993(00)03155-3. 11172772

[R3] Almaguer-Mederos LE, Aguilera-Rodríguez R, Almaguer-Gotay D, Hechavarría-Barzaga K, Álvarez-Sosa A, Chapman-Rodríguez Y, Silva-Ricardo Y, Gonźalez-Zaldivar Y, Vázquez-Mojena Y, Cuello-Almarales D, Rodríguez-Estupiñán A, 2020. Testosterone levels are decreased and associated with disease duration in male spinocerebellar ataxia type 2 patients. The Cerebellum 4 10.1007/s12311-020-01134-6. 32440846

[R4] Almey A, Milner TA, Brake WG, 2015. Estrogen receptors in the central nervous system and their implication for dopamine-dependent cognition in females. Horm Behav 10.1016/j.yhbeh.2015.06.010. PMC4820286 26122294

[R5] Amateau SK, Alt JJ, Stamps CL, McCarthy MM, 2004. Brain estradiol content in newborn rats: sex differences, regional heterogeneity, and possible de novo synthesis by the female telencephalon. Endocrinology 6. 10.1210/en.2003-1363. 14988386

[R6] Andreescu CE, Milojkovic BA, Haasdijk ED, Kramer P, De Jong FH, Krust A, De Zeeuw CI, De Jeu MT, 2007. Estradiol improves cerebellar memory formation by activating estrogen receptor β. J. Neurosci 40, doi. 10.1523/JNEUROSCI.2588-07.2007 PMC6672828 17913916

[R7] Arai Y, Matsumoto A, 1978. Synapse formation of the hypothalamic arcuate nucleus during post-natal development in the female rat and its modification by neonatal estrogen treatment. Psychoneuroendocrinology 1 10.1016/0306-4530(78)90039-2. 644014

[R8] Arao Y, Hamilton KJ, Lierz SL, Korach KS, 2018. N-terminal transactivation function, AF-1, of estrogen receptor alpha controls obesity through enhancement of energy expenditure. Mol Metab 10.1016/j.molmet.2018.09.006. PMC6308972 30287090

[R9] Argente J, Chowen-Breed JA, Steiner RA, Clifton DK, 1990. Somatostatin messenger RNA in hypothalamic neurons is increased by testosterone through activation of androgen receptors and not by aromatization to estradiol. Neuroendocrinology 4. 10.1159/000125618. 1979839

[R10] Atwi S, McMahon D, Scharfman H, MacLusky NJ, 2014. Androgen modulation of hippocampal structure and function. The Neuroscientist 1 10.1177/1073858414558065. PMC5002217 25416742

[R11] Austin D, Hamilton N, Elshimali Y, Pietras R, Wu Y, Vadgama J, 2018. Estrogen receptor-beta is a potential target for triple negative breast cancer treatment. Oncotarget 74 10.18632/oncotarget.26089. PMC6188058 30338035

[R12] Baez-Jurado E, Rincón-Benavides MA, Hidalgo-Lanussa O, Guio-Vega G, Ashraf GM, Sahebkar A, Echeverria V, Garcia-Segura LM, Barreto GE, 2019. Molecular mechanisms involved in the protective actions of selective estrogen receptor modulators in brain cells. Front. Neuroendocrinol 10.1016/j.yfrne.2018.09.001. 30223003

[R13] Bagamasbad P, Denver RJ, 2011. Mechanisms and significance of nuclear receptor auto- and cross-regulation. Gen. Comp. Endocrinol 1 10.1016/j.ygcen.2010.03.013. PMC2911511 20338175

[R14] Bálint M, Jeszenői N, Horváth I, Ábrahám IM, Hetényi C, 2017. Dynamic changes in binding interaction networks of sex steroids establish their non-classical effects. Sci. Rep 1 10.1038/s41598-017-14840-9. PMC5665952 29093525

[R15] Baron S, Manin M, Beaudoin C, Leotoing L, Communal Y, Veyssiere G, Morel L, 2004. Androgen receptor mediates non-genomic activation of phosphatidylinositol 3-OH kinase in androgen-sensitive epithelial cells. J. Biol. Chem 15 10.1074/jbc.M306143200. 14668339

[R16] Barone I, Brusco L, Fuqua SAW, 2010. Estrogen receptor mutations and changes in downstream gene expression and signaling. Clin. Can. Res.: Off. J. Am. Assoc. Can. Res 10 10.1158/1078-0432.CCR-09-1753. PMC4477803 20427689

[R17] Baumler E, Strickland L, Privitera L, 2019. Molecular Underpinnings of Estradiol-Mediated Sexual Dimorphism of Synaptic Plasticity in the Hippocampus of Rodents. J Neurosci 12 10.1523/JNEUROSCI.2894-18.2019. PMC6433759 30894462

[R18] Bean LA, Ianov L, Foster TC, 2014. Estrogen receptors, the hippocampus, and memory. The Neuroscientist 5, doi. 10.1177/1073858413519865 PMC4317255 24510074

[R19] Belcher SM, 1999. Regulated expression of estrogen receptor α and β mRNA in granule cells during development of the rat cerebellum. Dev. Brain Res 1 10.1016/S0165-3806(99)00050-4. 10366703

[R20] Belsham DD, Evangelou A, Roy D, Duc VL, Brown TJ, 1998. Regulation of gonadotropin-releasing hormone (GnRH) gene expression by 5alpha-dihydrotestosterone in GnRH-secreting GT1–7 hypothalamic neurons. Endocrinology 3. 10.1210/endo.139.3.5846. 9492044

[R21] Belsham DD, Lovejoy DA, 2005. Gonadotropin-releasing hormone: gene evolution, expression, and regulation. Vitam Horm 10.1016/S0083-6729(05)71003-7. 16112265

[R22] Beltramini M, Zambenedetti P, Wittkowski W, Zatta P, 2004. Effects of steroid hormones on the Zn, Cu and MTI/II levels in the mouse brain. Brain Res 1 10.1016/j.brainres.2004.04.010. 15196976

[R23] Berg AH, Rice CD, Rahman MS, Dong J, Thomas P, 2014. Identification and characterization of membrane androgen receptors in the ZIP9 zinc transporter subfamily: I. Discovery in female atlantic croaker and evidence ZIP9 mediates testosterone-induced apoptosis of ovarian follicle cells. Endocrinology 11. 10.1210/en.2014-1198. PMC4197986 25014354

[R24] Bermond B, Mos J, Meelis W, van der Poel AM, Kruk MR, 1982. Aggression induced by stimulation of the hypothalamus: Effects of androgens. Pharmacol. Biochem. Behav 1 10.1016/0091-3057(82)90010-7. 7036190

[R25] Beyer C, Green SJ, Hutchison JB, 1994. Androgens influence sexual differentiation of embryonic mouse hypothalamic aromatase neurons in vitro. Endocrinology 3. 10.1210/endo.135.3.8070366. 8070366

[R26] Beyer C, Hutchison JB, 1997. Androgens stimulate the morphological maturation of embryonic hypothalamic aromatase-immunoreactive neurons in the mouse. Dev. Brain Res 1 10.1016/S0165-3806(96)00170-8. 9027406

[R27] Bhatia A, Sekhon HK, Kaur G, 2014. Sex hormones and immune dimorphism. ScientificWorldJournal 10.1155/2014/159150. PMC4251360 25478584

[R28] Bian C, Bai B, Gao Q, Li S, Zhao Y, 2019a. 17beta-estradiol regulates glucose metabolism and insulin secretion in rat islet beta cells through GPER and Akt/mTOR/GLUT2 pathway. Front Endocrinol (Lausanne) 10.3389/fendo.2019.00531. PMC6691154 31447779

[R29] Bian C, Bai B, Gao Q, Li S, Zhao Y, 2019b. 17β-estradiol regulates glucose metabolism and insulin secretion in Rat Islet β cells through GPER and Akt/mTOR/GLUT2 pathway. Front. Endocrinol 10.3389/fendo.2019.00531. PMC6691154 31447779

[R30] Bondesson M, Hao R, Lin C-Y, Williams C, Gustafsson J-Å, 2015. Estrogen receptor signaling during vertebrate development. BBA 2. 10.1016/j.bbagrm.2014.06.005. PMC4269570 24954179

[R31] Bourguet W, Germain P, Gronemeyer H, 2000. Nuclear receptor ligand-binding domains: three-dimensional structures, molecular interactions and pharmacological implications. Trends Pharmacol. Sci 10 10.1016/S0165-6147(00)01548-0. 11050318

[R32] Bowers JM, Waddell J, McCarthy M, 2010. A developmental sex difference in hippocampal neurogenesis is mediated by endogenous oestradiol. Biology of sex differences 10.1186/2042-6410-1-8. PMC3016241 21208470

[R33] Brisken C, O’Malley B, 2010. Hormone action in the mammary gland. Cold Spring Harbor Perspect. Biol 12 10.1101/cshperspect.a003178. PMC2982168 20739412

[R34] Bruchovsky N, Wilson JD, 1968a. The conversion of testosterone to 5-alpha-androstan-17-beta-ol-3-one by rat prostate in vivo and in vitro. J Biol Chem 8, doi. 4384673

[R35] Bruchovsky N, Wilson JD, 1968b. The intranuclear binding of testosterone and 5-alpha-androstan-17-beta-ol-3-one by rat prostate. J Biol Chem 22, doi. 5696629

[R36] Bulldan A, Bartsch JW, Konrad L, Scheiner-Bobis G, 2018. ZIP9 but not the androgen receptor mediates testosterone-induced migratory activity of metastatic prostate cancer cells. Biochim Biophys Acta Mol Cell Res 12 10.1016/j.bbamcr.2018.09.004. 30262433

[R37] Bulldan A, Dietze R, Shihan M, Scheiner-Bobis G, 2016. Non-classical testosterone signaling mediated through ZIP9 stimulates claudin expression and tight junction formation in Sertoli cells. Cell. Signal 8 10.1016/j.cellsig.2016.04.015. 27164415

[R38] Burdakov D, Peleg-Raibstein D, 2020. The hypothalamus as a primary coordinator of memory updating. Physiol. Behav 10.1016/j.physbeh.2020.112988. 32485184

[R39] Burstein SR, Kim HJ, Fels JA, Qian L, Zhang S, Zhou P, Starkov AA, Iadecola C, Manfredi G, 2018. Estrogen receptor beta modulates permeability transition in brain mitochondria. Biochim Biophys Acta Bioenerg 6 10.1016/j.bbabio.2018.03.006. PMC5912174 29550215

[R40] Cabrera Zapata LE, Bollo M, Cambiasso MJ, 2019. Estradiol-Mediated Axogenesis of Hypothalamic Neurons Requires ERK1/2 and Ryanodine Receptors-Dependent Intracellular Ca(2+) Rise in Male Rats. Front. Cell. Neurosci 10.3389/fncel.2019.00122. PMC6454002 31001087

[R41] Campbell M, Jialal I, 2020. Physiology. Endocrine Hormones, StatPearls, Treasure Island (FL). 30860733

[R42] Carmeci C, Thompson DA, Ring HZ, Francke U, Weigel RJ, 1997. Identification of a Gene (GPR30) with Homology to the G-Protein-Coupled Receptor Superfamily Associated with Estrogen Receptor Expression in Breast Cancer. Genomics 3. 10.1006/geno.1997.4972. 9367686

[R43] Carney RSE, 2019. Concurrent Medial Prefrontal Cortex and Dorsal Hippocampal Activity Is Required for Estradiol-Mediated Effects on Object Memory and Spatial Memory Consolidation. eNeuro 4, doi: 10.1523/ENEURO.0271-19.2019. PMC6709231 31431561

[R44] Carrier N, Saland SK, Duclot F, He H, Mercer R, Kabbaj M, 2015. The anxiolytic and antidepressant-like effects of testosterone and estrogen in gonadectomized male rats. Biol. Psychiatry 4. 10.1016/j.biopsych.2014.12.024. PMC4501899 25683735

[R45] Carson JA, Manolagas SC, 2015. Effects of sex steroids on bones and muscles: similarities, parallels, and putative interactions in health and disease. Bone 10.1016/j.bone.2015.04.015. PMC4600533 26453497

[R46] Celotti F, Negri-Cesi P, Poletti A, 1997. Steroid metabolism in the mammalian brain: 5alpha-reduction and aromatization. Brain Res. Bull 4, doi. 10.1016/s0361-9230(97)00216-5 9370201

[R47] Cisternas CD, Cabrera Zapata LE, Mir FR, Scerbo MJ, Arevalo MA, Garcia-Segura LM, Cambiasso MJ, 2020. Estradiol-dependent axogenesis and Ngn3 expression are determined by XY sex chromosome complement in hypothalamic neurons. Sci. Rep 1 10.1038/s41598-020-65183-x. PMC7237695 32427857

[R48] Clipperton AE, Spinato JM, Chernets C, Pfaff DW, Choleris E, 2008. Differential effects of estrogen receptor alpha and beta specific agonists on social learning of food preferences in female mice. Neuropsychopharmacology 10 10.1038/sj.npp.1301625. 18004284

[R49] Colciago A, Celotti F, Pravettoni A, Mornati O, Martini L, Negri-Cesi P, 2005. Dimorphic expression of testosterone metabolizing enzymes in the hypothalamic area of developing rats. Dev. Brain Res 2 10.1016/j.devbrainres.2004.12.003. 15804399

[R50] Cooke B, Hegstrom CD, Villeneuve LS, Breedlove SM, 1998. Sexual differentiation of the vertebrate brain: principles and mechanisms. Front. Neuroendocrinol 4, doi. 10.1006/frne.1998.0171 9799588

[R51] Crimins JL, Wang AC-J, Yuk F, Puri R, Janssen WGM, Hara Y, Rapp PR, Morrison JH, 2016. Diverse synaptic distributions of g protein-coupled estrogen receptor 1 in monkey prefrontal cortex with aging and menopause. Cereb. Cortex 3. 10.1093/cercor/bhw050. PMC5909633 26941383

[R52] Crowley S, 2017. Exercise, depression-anxiety disorders and sex. Hormones 171–191.28742505

[R53] Davey RA, Clarke MV, Russell PK, Rana K, Seto J, Roeszler KN, How JMY, Chia LY, North K, Zajac JD, 2017. Androgen action via the androgen receptor in neurons within the brain positively regulates muscle mass in male mice. Endocrinology 10. 10.1210/en.2017-00470. 28977603

[R54] Day JR, Frank AT, O’Callaghan JP, Jones BC, Anderson JE, 1998. The effect of age and testosterone on the expression of glial fibrillary acidic protein in the rat cerebellum. Exp. Neurol 2 10.1006/exnr.1998.6801. 9628769

[R55] Debes JD, Tindall DJ, 2002. The role of androgens and the androgen receptor in prostate cancer. Cancer Lett 1–2 10.1016/s0304-3835(02)00413-5. 12359344

[R56] Denley MCS, Gatford NJF, Sellers KJ, Srivastava DP, 2018. Estradiol and the development of the cerebral cortex: an unexpected role? Front. Neurosci 10.3389/fnins.2018.00245. PMC5981095 29887794

[R57] Deschamps AM, Murphy E, 2009. Activation of a novel estrogen receptor, GPER, is cardioprotective in male and female rats. Am J Physiol Heart Circ Physiol 5 10.1152/ajpheart.00283.2009. PMC2781389 19717735

[R58] Díaz-Sánchez V, Morimoto S, Morales A, Robles-Díaz G, Cerbón M, 1995. Androgen receptor in the rat pancreas: genetic expression and steroid regulation. Pancreas 3, doi. 10.1097/00006676-199510000-00005 8577677

[R59] Dieni CV, Contemori S, Biscarini A, Panichi R, 2020. De Novo synthesized estradiol: a role in modulating the cerebellar function. Int. J. Mol. Sci 9 10.3390/ijms21093316. PMC7247543 32392845

[R60] Dieni CV, Sullivan JA, Faralli M, Contemori S, Biscarini A, Pettorossi VE, Panichi R, 2018. 17 beta-estradiol synthesis modulates cerebellar dependent motor memory formation in adult male rats. Neurobiol. Learn. Mem 10.1016/j.nlm.2018.08.011. 30125696

[R61] Dieudonne M, Pecquery R, Boumediene A, Leneveu M, Giudicelli Y, 1998. Androgen receptors in human preadipocytes and adipocytes: regional specificities and regulation by sex steroids. Am. J. Physiol.-Cell Physiol 6, doi. 10.1152/ajpcell.1998.274.6.C1645 9611130

[R62] Diotel N, Charlier TD, Lefebvre d’Hellencourt C, Couret D, Trudeau VL, Nicolau JC, Meilhac O, Kah O, Pellegrini E, 2018. Steroid Transport, Local Synthesis, and Signaling within the Brain: Roles in Neurogenesis, Neuroprotection, and Sexual Behaviors. Front Neurosci 10.3389/fnins.2018.00084. PMC5826223 29515356

[R63] Dominguez R, Zitting M, Liu Q, Patel A, Babadjouni R, Hodis DM, Chow RH, Mack WJ, 2018. Estradiol Protects White Matter of Male C57BL6J Mice against Experimental Chronic Cerebral Hypoperfusion. J Stroke Cerebrovasc Dis 7 10.1016/j.jstrokecerebrovasdis.2018.01.030. PMC5972054 29602614

[R64] dos Santos RL, da Silva FB, Ribeiro RF Jr., Stefanon I, 2014. Sex hormones in the cardiovascular system. Horm Mol Biol Clin Investig 2 10.1515/hmbci-2013-0048. 25390005

[R65] Duarte-Guterman P, Yagi S, Chow C, Galea LA, 2015. Hippocampal learning, memory, and neurogenesis: Effects of sex and estrogens across the lifespan in adults. Horm Behav 10.1016/j.yhbeh.2015.05.024. 26122299

[R66] Duarte AC, Hrynchak M, GonÁalves I, Quintela T, and Santos C, 2016. Sex Hormone Decline and Amyloid ≤ Synthesis, Transport and Clearance in the Brain. Journal of neuroendocrinology10.1111/jne.12432 27632792

[R67] Evans PD, 2019. Rapid signalling responses via the G protein-coupled estrogen receptor, GPER, in a hippocampal cell line. Steroids 10.1016/j.steroids.2019.108487. 31499073

[R68] Fattoretti P, Malatesta M, Mariotti R, Zancanaro C, 2019. Testosterone administration increases synaptic density in the gyrus dentatus of old mice independently of physical exercise. Exp. Gerontol 10.1016/j.exger.2019.110664. 31319132

[R69] Filardo E, Quinn J, Pang Y, Graeber C, Shaw S, Dong J, Thomas P, 2007. Activation of the novel estrogen receptor G protein-coupled receptor 30 (GPR30) at the plasma membrane. Endocrinology 7. 10.1210/en.2006-1605. 17379646

[R70] Filardo EJ, Quinn JA, Bland KI, Frackelton AR Jr., 2000. Estrogen-induced activation of Erk-1 and Erk-2 requires the G protein-coupled receptor homolog, GPR30, and occurs via trans-activation of the epidermal growth factor receptor through release of HB-EGF. Mol Endocrinol 10 10.1210/mend.14.10.0532. 11043579

[R71] Fındıklı E, Camkurt MA, Karaaslan MF, Kurutas EB, Altun H, İzci F, Fındıklı HA, Kardas S, 2016. Serum levels of G protein-coupled estrogen receptor 1 (GPER1) in drug-naive patients with generalized anxiety disorder. Psychiatry Res 10.1016/j.psychres.2016.04.098. 27512921

[R72] Fleischer AW, Schalk JC, Wetzel EA, Hanson AM, Sem DS, Donaldson WA, Frick KM, 2020. Chronic oral administration of a novel estrogen receptor beta agonist enhances memory consolidation and alleviates vasomotor symptoms in a mouse model of menopause. Alzheimer’s & Dementia S9 10.1002/alz.047645. PMC8680219 33571507

[R73] Fortress AM, Kim J, Poole RL, Gould TJ, Frick KM, 2014. 17β-Estradiol regulates histone alterations associated with memory consolidation and increases Bdnf promoter acetylation in middle-aged female mice. Learning & Memory 9, doi. 10.1101/lm.034033.113 PMC4138358 25128537

[R74] Foster TC, 2012. Role of estrogen receptor alpha and beta expression and signaling on cognitive function during aging. Hippocampus 4 10.1002/hipo.20935. PMC3704216 21538657

[R75] Frankiensztajn LM, Elliott E, Koren O, 2020. The microbiota and the hypothalamus-pituitary-adrenocortical (HPA) axis, implications for anxiety and stress disorders. Curr. Opin. Neurobiol 10.1016/j.conb.2019.12.003. 31972462

[R76] Frick KM, 2015. Molecular mechanisms underlying the memory-enhancing effects of estradiol. Horm. Behav 10.1016/j.yhbeh.2015.05.001. PMC4573242 25960081

[R77] Frick KM, Kim J, 2018. Mechanisms underlying the rapid effects of estradiol and progesterone on hippocampal memory consolidation in female rodents. Horm Behav 10.1016/j.yhbeh.2018.04.013. PMC6226372 29727606

[R78] Frick KM, Kim J, Tuscher JJ, Fortress AM, 2015. Sex steroid hormones matter for learning and memory: estrogenic regulation of hippocampal function in male and female rodents. Learn Mem 9 10.1101/lm.037267.114. PMC4561402 26286657

[R79] Fridolfsson HN, Roth DM, Insel PA, Patel HH, 2014. Regulation of intracellular signaling and function by caveolin. FASEB J 9 10.1096/fj.14-252320. PMC4139902 24858278

[R80] Galea LAM, Roes MM, Dimech CJ, Chow C, Mahmoud R, Lieblich SE, Duarte-Guterman P, 2018. Premarin has opposing effects on spatial learning, neural activation, and serum cytokine levels in middle-aged female rats depending on reproductive history. Neurobiol Aging 10.1016/j.neurobiolaging.2018.06.030. 30056312

[R81] Gibbs RB, Gabor R, 2003. Estrogen and cognition: applying preclinical findings to clinical perspectives. J Neurosci Res 5 10.1002/jnr.10811. 14635215

[R82] Giovannelli P, Di Donato M, Galasso G, Di Zazzo E, Bilancio A, Migliaccio A, 2018. The Androgen Receptor in Breast Cancer. Front. Endocrinol 10.3389/fendo.2018.00492. PMC6122126 30210453

[R83] Gohar EY, Daugherty EM, Aceves JO, Sedaka R, Obi IE, Allan JM, Soliman RH, Jin C, De Miguel C, Lindsey SH, Pollock JS, Pollock DM, 2020. Evidence for G-Protein-Coupled Estrogen Receptor as a Pronatriuretic Factor. J Am Heart Assoc 10 10.1161/JAHA.119.015110. PMC7660860 32390531

[R84] González-Granillo M, Savva C, Li X, Ghosh Laskar M, Angelin B, Gustafsson J-Å, Korach-André M, 2020. Selective estrogen receptor (ER)β activation provokes a redistribution of fat mass and modifies hepatic triglyceride composition in obese male mice. Mol. Cell. Endocrinol 10.1016/j.mce.2019.110672. 31811898

[R85] Gonzalez-Granillo M, Savva C, Li X, Ghosh Laskar M, Angelin B, Gustafsson JA, Korach-Andre M, 2020. Selective estrogen receptor (ER)beta activation provokes a redistribution of fat mass and modifies hepatic triglyceride composition in obese male mice. Mol Cell Endocrinol 10.1016/j.mce.2019.110672. 31811898

[R86] González M, Cabrera-Socorro A, Pérez-García CG, Fraser JD, López FJ, Alonso R, Meyer G, 2007. Distribution patterns of estrogen receptor α and β in the human cortex and hippocampus during development and adulthood. Journal of Comparative Neurology 6 10.1002/cne.21419. 17570500

[R87] Gorelick DA, Watson W, Halpern ME, 2008. Androgen receptor gene expression in the developing and adult zebrafish brain. Dev. Dyn 10 10.1002/dvdy.21700. 18816841

[R88] Green MR, Zeidan M, Hodges TE, McCormick CM, 2019. Age-dependent regulation by androgens of gene expression in the anterior hypothalamus and stress-induced release of adrenal hormones in adolescent and adult male rats. J. Neuroendocrinol 6 10.1111/jne.12714. 30912177

[R89] Griswold MD, 1998. The central role of Sertoli cells in spermatogenesis. Semin. Cell Dev. Biol 4 10.1006/scdb.1998.0203. 9813187

[R90] Gubbels Bupp MR, Jorgensen TN, 2018. Androgen-Induced Immunosuppression. Front Immunol 10.3389/fimmu.2018.00794. PMC5932344 29755457

[R91] Guo G, Kang L, Geng D, Han S, Li S, Du J, Wang C, Cui H, 2020. Testosterone modulates structural synaptic plasticity of primary cultured hippocampal neurons through ERK - CREB signalling pathways. Mol. Cell. Endocrinol 10.1016/j.mce.2019.110671. 31805308

[R92] Hamilton DJ, Minze LJ, Kumar T, Cao TN, Lyon CJ, Geiger PC, Hsueh WA, Gupte AA, 2016. Estrogen receptor alpha activation enhances mitochondrial function and systemic metabolism in high-fat-fed ovariectomized mice. Physiol Rep 17 10.14814/phy2.12913. PMC5027347 27582063

[R93] Hammond R, Mauk R, Ninaci D, Nelson D, Gibbs RB, 2009. Chronic treatment with estrogen receptor agonists restores acquisition of a spatial learning task in young ovariectomized rats. Horm. Behav 3 10.1016/j.yhbeh.2009.06.008. PMC2772993 19560466

[R94] Hampson E, 2018. Estrogens, Aging, and Working Memory. Current Psychiatry Reports 12 10.1007/s11920-018-0972-1. PMC6182645 30306352

[R95] Hamson D, Wainwright S, Taylor J, Jones B, Watson N, Galea L, 2013. Androgens Increase Survival of Adult-Born Neurons in the Dentate Gyrus by an Androgen Receptor-Dependent Mechanism in Male Rats. Endocrinology 10.1210/en.2013-1129. 23782943

[R96] Hanson AM, Perera KLIS, Kim J, Pandey RK, Sweeney N, Lu X, Imhoff A, Mackinnon AC, Wargolet AJ, Van Hart RM, Frick KM, Donaldson WA, Sem DS, 2018. A-C Estrogens as Potent and Selective Estrogen Receptor-Beta Agonists (SERBAs) to Enhance Memory Consolidation under Low-Estrogen Conditions. J. Med. Chem 11 10.1021/acs.jmedchem.7b01601. PMC7217030 29741891

[R97] Hashikawa K, Hashikawa Y, Tremblay R, Zhang J, Feng JE, Sabol A, Piper WT, Lee H, Rudy B, Lin D, 2017. Esr1+ cells in the ventromedial hypothalamus control female aggression. Nat. Neurosci 11 10.1038/nn.4644. PMC5953764 28920934

[R98] Hauger LE, Westlye LT, Fjell AM, Walhovd KB, Bjørnebekk A, 2019. Structural brain characteristics of anabolic–androgenic steroid dependence in men. Addiction 8 10.1111/add.14629. PMC6767448 30955206

[R99] Hazell GGJ, Yao ST, Roper JA, Prossnitz ER, O’Carroll A-M, Lolait SJ, 2009. Localisation of GPR30, a novel G protein-coupled oestrogen receptor, suggests multiple functions in rodent brain and peripheral tissues. The Journal of endocrinology 2 10.1677/JOE-09-0066. PMC2710976 19420011

[R100] Hedges VL, Chen G, Yu L, Krentzel AA, Starrett JR, Zhu J-N, Suntharalingam P, Remage-Healey L, Wang J-J, Ebner TJ, Mermelstein PG, 2018. Local Estrogen Synthesis Regulates Parallel Fiber-Purkinje Cell Neurotransmission Within the Cerebellar Cortex. Endocrinology 3. 10.1210/en.2018-00039. PMC5839732 29381778

[R101] Heemers HV, Tindall DJ, 2007. Androgen receptor (AR) coregulators: a diversity of functions converging on and regulating the AR transcriptional complex. Endocr Rev 7 10.1210/er.2007-0019. 17940184

[R102] Heinlein CA, Chang C, 2002. The roles of androgen receptors and androgen-binding proteins in nongenomic androgen actions. Mol Endocrinol 10 10.1210/me.2002-0070. 12351684

[R103] Hoffman JF, Wright CL, McCarthy MM, 2016. A critical period in Purkinje cell development is mediated by local estradiol synthesis, disrupted by inflammation, and has enduring consequences only for males. J. Neurosci 39, doi. 10.1523/JNEUROSCI.1262-16.2016 PMC5039254 27683901

[R104] Hough D, Bellingham M, Haraldsen IRH, McLaughlin M, Rennie M, Robinson JE, Solbakk AK, Evans NP, 2017. Spatial memory is impaired by peripubertal GnRH agonist treatment and testosterone replacement in sheep. Psychoneuroendocrinology 10.1016/j.psyneuen.2016.10.016. PMC5140006 27837697

[R105] Hsu H-H, Cheng S-F, Wu C-C, Chu C-H, Weng Y-J, Lin C-S, Lee S-D, Wu H-C, Huang C-Y, and Kuo W-W, 2006. Apoptotic effects of over-expressed estrogen receptor-β on LoVo colon cancer cell is mediated by p53 signalings in a ligand-dependent manner. The Chinese journal of physiology 16830793

[R106] Hutson DD, Gurrala R, Ogola BO, Zimmerman MA, Mostany R, Satou R, Lindsey SH, 2019. Estrogen receptor profiles across tissues from male and female Rattus norvegicus. Biology of sex. Differences 1, doi. 10.1186/s13293-019-0219-9 PMC6329134 30635056

[R107] Chen CV, Brummet JL, Lonstein JS, Jordan CL, Breedlove SM, 2014. New knockout model confirms a role for androgen receptors in regulating anxiety-like behaviors and HPA response in mice. Horm. Behav 3 10.1016/j.yhbeh.2014.01.001. PMC4295784 24440052

[R108] Chhibber A, Woody SK, Karim Rumi MA, Soares MJ, Zhao L, 2017. Estrogen receptor β deficiency impairs BDNF-5-HT(2A) signaling in the hippocampus of female brain: A possible mechanism for menopausal depression. Psychoneuroendocrinology 10.1016/j.psyneuen.2017.05.016. PMC5523821 28544903

[R109] Chimento A, Sirianni R, Casaburi I, Pezzi V, 2014. Role of estrogen receptors and g protein-coupled estrogen receptor in regulation of hypothalamus-pituitary-testis axis and spermatogenesis. Front Endocrinol (Lausanne) 10.3389/fendo.2014.00001. PMC3893621 24474947

[R110] Chung YS, Poppe A, Novotny S, Epperson CN, Kober H, Granger DA, Blumberg HP, Ochsner K, Gross JJ, Pearlson G, Stevens MC, 2019. A preliminary study of association between adolescent estradiol level and dorsolateral prefrontal cortex activity during emotion regulation. Psychoneuroendocrinology 10.1016/j.psyneuen.2019.104398. PMC6842698 31394491

[R111] Ikeda K, Horie-Inoue K, Inoue S, 2015. Identification of estrogen-responsive genes based on the DNA binding properties of estrogen receptors using high-throughput sequencing technology. Acta Pharmacol. Sin 1 10.1038/aps.2014.123. PMC4571320 25500870

[R112] Ikeda K, Inoue S, 2004. Estrogen receptors and their downstream targets in cancer. Arch. Histol. Cytol 5, doi. 10.1679/aohc.67.435 15781984

[R113] Ikeda Y, Aihara K, Sato T, Akaike M, Yoshizumi M, Suzaki Y, Izawa Y, Fujimura M, Hashizume S, Kato M, Yagi S, Tamaki T, Kawano H, Matsumoto T, Azuma H, Kato S, Matsumoto T, 2005. Androgen receptor gene knockout male mice exhibit impaired cardiac growth and exacerbation of angiotensin II-induced cardiac fibrosis. J Biol Chem 33 10.1074/jbc.M411694200. 15961403

[R114] Iorga A, Umar S, Ruffenach G, Aryan L, Li J, Sharma S, Motayagheni N, Nadadur RD, Bopassa JC, Eghbali M, 2018. Estrogen rescues heart failure through estrogen receptor Beta activation. Biology of sex differences 1 10.1186/s13293-018-0206-6. PMC6208048 30376877

[R115] Iravani M, Lagerquist M, Ohlsson C, Savendahl L, 2017. Regulation of bone growth via ligand-specific activation of estrogen receptor alpha. J Endocrinol 3 10.1530/JOE-16-0263. 27999091

[R116] Islam MN, Sakimoto Y, Jahan MR, Ishida M, Tarif AMM, Nozaki K, Masumoto K-H, Yanai A, Mitsushima D, Shinoda K, 2020. Androgen Affects the Dynamics of Intrinsic Plasticity of Pyramidal Neurons in the CA1 Hippocampal Subfield in Adolescent Male Rats. Neuroscience 10.1016/j.neuroscience.2020.05.025. 32450298

[R117] Islam MN, Sakimoto Y, Jahan MR, Miyasato E, Tarif AMM, Nozaki K, Masumoto K-H, Yanai A, Mitsushima D, Shinoda K, 2021. Androgen Affects the Inhibitory Avoidance Memory by Primarily Acting on Androgen Receptor in the Brain in Adolescent Male Rats. Brain Sciences 2, doi. 10.3390/brainsci11020239 PMC7918178 33672867

[R118] Ivanova T, Karolczak M, Beyer C, 2002. Estradiol Stimulates GDNF Expression in Developing Hypothalamic Neurons. Endocrinology 8. 10.1210/endo.143.8.8794. 12130584

[R119] Jacobson W, Routledge J, Davies H, Saich T, Hughes I, Brinkmann A, Brown B, Clarkson P, 1995. Localisation of androgen receptors in dermal fibroblasts, grown in vitro, from normal subjects and from patients with androgen insensitivity syndrome. Horm Res 2 10.1159/000184598. 7590636

[R120] Jakab RL, Wong JK, Belcher SM, 2001. Estrogen receptor β immunoreactivity in differentiating cells of the developing rat cerebellum. Journal of Comparative Neurology 3 10.1002/1096-9861(20010212)430:3<396::AID-CNE1039>3.0.CO;2-0. 11169476

[R121] Jelks KB, Wylie R, Floyd CL, McAllister AK, Wise P, 2007. Estradiol targets synaptic proteins to induce glutamatergic synapse formation in cultured hippocampal neurons: critical role of estrogen receptor-α. J. Neurosci 26, doi. 10.1523/JNEUROSCI.0909-07.2007 PMC6672227 17596438

[R122] Jensen E, 2012. A conversation with Elwood Jensen. Interview by David D. Moore. Annu Rev Physiol doi: 10.1146/annurev-physiol-020911-153327. 21888507

[R123] Jiang M, Ma X, Zhao Q, Li Y, Xing Y, Deng Q, Shen Y, 2019. The neuroprotective effects of novel estrogen receptor GPER1 in mouse retinal ganglion cell degeneration. Exp. Eye Res 10.1016/j.exer.2019.107826. 31586450

[R124] Jin H-J, Kim J, Yu J, 2013. Androgen receptor genomic regulation. Translational andrology and urology 3 10.3978/j.issn.2223-4683.2013.09.01. PMC4165347 25237629

[R125] Kalyvianaki K, Panagiotopoulos AA, Malamos P, Moustou E, Tzardi M, Stathopoulos EN, Ioannidis GS, Marias K, Notas G, Theodoropoulos PA, Castanas E, Kampa M, 2019. Membrane androgen receptors (OXER1, GPRC6A AND ZIP9) in prostate and breast cancer: A comparative study of their expression. Steroids 10.1016/j.steroids.2019.01.006. 30707908

[R126] Kaminska A, Pardyak L, Marek S, Wrobel K, Kotula-Balak M, Bilinska B, Hejmej A, 2020. Notch signaling regulates nuclear androgen receptor AR and membrane androgen receptor ZIP9 in mouse Sertoli cells. Andrology 2 10.1111/andr.12691. 31468707

[R127] Kang HY, Cho CL, Huang KL, Wang JC, Hu YC, Lin HK, Chang C, Huang KE, 2004. Nongenomic androgen activation of phosphatidylinositol 3-kinase/Akt signaling pathway in MC3T3-E1 osteoblasts. J Bone Miner Res 7 10.1359/JBMR.040306. 15177002

[R128] Kansra S, Yamagata S, Sneade L, Foster L, Ben-Jonathan N, 2005. Differential effects of estrogen receptor antagonists on pituitary lactotroph proliferation and prolactin release. Mol Cell Endocrinol 1–2 10.1016/j.mce.2005.04.008. 15950373

[R129] Karaismailoğlu S, Erdem A, 2013. The effects of prenatal sex steroid hormones on sexual differentiation of the brain. J Turk Ger Gynecol Assoc 3 10.5152/jtgga.2013.86836. PMC3928415 24592097

[R130] Kashkin KB, Kleber HD, 1989. Hooked on hormones? An anabolic steroid addiction hypothesis. JAMA 22 10.1001/jama.262.22.3166. 2681859

[R131] Kastenberger I, Schwarzer C, 2014. GPER1 (GPR30) knockout mice display reduced anxiety and altered stress response in a sex and paradigm dependent manner. Horm. Behav 4 10.1016/j.yhbeh.2014.09.001. PMC4213071 25236887

[R132] Kim J, Schalk JC, Koss WA, Gremminger RL, Taxier LR, Gross KS, Frick KM, 2019a. Dorsal Hippocampal Actin Polymerization Is Necessary for Activation of G-Protein-Coupled Estrogen Receptor (GPER) to Increase CA1 Dendritic Spine Density and Enhance Memory Consolidation. The Journal of neuroscience : the official journal of the Society for Neuroscience 48 10.1523/JNEUROSCI.2687-18.2019. PMC6880457 31628182

[R133] Kim J, Schalk JC, Koss WA, Gremminger RL, Taxier LR, Gross KS, Frick KM, 2019b. Dorsal Hippocampal Actin Polymerization Is Necessary for Activation of G-Protein-Coupled Estrogen Receptor (GPER) to Increase CA1 Dendritic Spine Density and Enhance Memory Consolidation. J. Neurosci 48, doi. 10.1523/JNEUROSCI.2687-18.2019 PMC6880457 31628182

[R134] Kim J, Szinte J, Iden M, Frick K, 2016a. 17 -Estradiol and Agonism of G-protein-Coupled Estrogen Receptor Enhance Hippocampal Memory via Different Cell-Signaling Mechanisms. J. Neurosci 10.1523/JNEUROSCI.0257-15.2016. PMC4792941 26985039

[R135] Kim J, Szinte JS, Boulware MI, Frick KM, 2016b. 17beta-Estradiol and Agonism of G-protein-Coupled Estrogen Receptor Enhance Hippocampal Memory via Different Cell-Signaling Mechanisms. J Neurosci 11 10.1523/JNEUROSCI.0257-15.2016. PMC4792941 26985039

[R136] Kim J, Szinte JS, Boulware MI, Frick KM, 2016c. 17β-Estradiol and Agonism of G-protein-Coupled Estrogen Receptor Enhance Hippocampal Memory via Different Cell-Signaling Mechanisms. The Journal of neuroscience : the official journal of the Society for Neuroscience 11 10.1523/JNEUROSCI.0257-15.2016. PMC4792941 26985039

[R137] King JA, Barkley RA, Delville Y, Ferris CF, 2000. Early androgen treatment decreases cognitive function and catecholamine innervation in an animal model of ADHD. Behav. Brain Res 1 10.1016/S0166-4328(99)00113-8. 10628728

[R138] Komrakova M, Furtwangler J, Hoffmann DB, Lehmann W, Schilling AF, Sehmisch S, 2020. The Selective Androgen Receptor Modulator Ostarine Improves Bone Healing in Ovariectomized Rats. Calcif Tissue Int 2 10.1007/s00223-019-00613-1. 31531719

[R139] Koss W, Haertel J, Philippi S, and Frick K, Sex differences in the rapid cell signaling mechanisms underlying the memory-enhancing effects of 17β-estradiol. eNeuro 5 (5), 2018a. 10.1523/ENEURO.0267-18.2018 PMC6220582 30406188

[R140] Koss WA, Frick KM, 2019. Activation of androgen receptors protects intact male mice from memory impairments caused by aromatase inhibition. Horm. Behav 10.1016/j.yhbeh.2019.01.002. PMC6527464 30653980

[R141] Koss WA, Haertel JM, Philippi SM, Frick KM, 2018b. Sex Differences in the Rapid Cell Signaling Mechanisms Underlying the Memory-Enhancing Effects of 17beta-Estradiol. eNeuro 5 10.1523/ENEURO.0267-18.2018. PMC6220582 30406188

[R142] Kotula-Balak M, Pawlicki P, Milon A, Tworzydlo W, Sekula M, Pacwa A, Gorowska-Wojtowicz E, Bilinska B, Pawlicka B, Wiater J, Zarzycka M, Galas J, 2018. The role of G-protein-coupled membrane estrogen receptor in mouse Leydig cell function—in vivo and in vitro evaluation. Cell Tissue Res 2 10.1007/s00441-018-2861-7. PMC6209072 29876633

[R143] Kousteni S, Bellido T, Plotkin LI, O’Brien CA, Bodenner DL, Han L, Han K, DiGregorio GB, Katzenellenbogen JA, Katzenellenbogen BS, Roberson PK, Weinstein RS, Jilka RL, Manolagas SC, 2001. Nongenotropic, sex-nonspecific signaling through the estrogen or androgen receptors: dissociation from transcriptional activity. Cell 5, doi. 11257226

[R144] Kovacs E, Maclusky N, Leranth C, 2003. Effects of testosterone on hippocampal CA1 spine synaptic density in the male are inhibited by fimbria/fornix transection. Neuroscience 10.1016/j.neuroscience.2003.08.046. 14622923

[R145] Krentzel AA, Barrett LR, Meitzen J, 2019. Estradiol rapidly modulates excitatory synapse properties in a sex- and region-specific manner in rat nucleus accumbens core and caudate-putamen. J Neurophysiol 3 10.1152/jn.00264.2019. PMC6766735 31314648

[R146] Krȩżel W, Dupont S, Krust A, Chambon P, Chapman PF, 2001. Increased anxiety and synaptic plasticity in estrogen receptor β-deficient mice. Proc. Natl. Acad. Sci 21 10.1073/pnas.221451898. PMC59805 11593044

[R147] Lange I, Daxenberger A, and Meyer HHD, 2001. Tissue-specific expression pattern of estrogen receptors (ER): Quantification of ER?? and ER?? mRNA with real-time RT-PCR. APMIS : acta pathologica, microbiologica, et immunologica Scandinavica10.1034/j.1600-0463.2001.090503.x 11478682

[R148] Lange I, Meyer H, 2003. The gastrointestinal tract as target of steroid hormone action: quantification of steroid receptor mRNA expression (AR, ERalpha, ERbeta and PR) in 10 bovine gastrointestinal tract compartments by kinetic RT-PCR. The Journal of steroid biochemistry and molecular biology 10.1016/S0960-0760(03)00025-6. 12710999

[R149] Lee C, Sutkowski D, Sensibar J, Zelner D, Kim I, Amsel I, Shaw N, Prins G, Kozlowski J, 1995. Regulation of proliferation and production of prostate-specific antigen in androgen-sensitive prostatic cancer cells, LNCaP, by dihydrotestosterone. Endocrinology 10.1210/en.136.2.796. 7530653

[R150] Leonetti D, Soleti R, Clere N, Vergori L, Jacques C, Duluc L, Dourguia C, Martinez MC, Andriantsitohaina R, 2018. Extract Enriched in Flavan-3-ols and Mainly Procyanidin Dimers Improves Metabolic Alterations in a Mouse Model of Obesity-Related Disorders Partially via Estrogen Receptor Alpha. Front Pharmacol 10.3389/fphar.2018.00406. PMC5928481 29740325

[R151] Leranth C, Hajszan T, MacLusky NJ, 2004. Androgens Increase Spine Synapse Density in the CA1 Hippocampal Subfield of Ovariectomized Female Rats. The Journal of Neuroscience 2 10.1523/JNEUROSCI.4516-03.2004. PMC6729992 14724248

[R152] Leranth C, Petnehazy O, MacLusky NJ, 2003. Gonadal hormones affect spine synaptic density in the CA1 hippocampal subfield of male rats. The Journal of neuroscience : the official journal of the Society for Neuroscience 5 10.1523/JNEUROSCI.23-05-01588.2003. PMC6741990 12629162

[R153] Leung JK, Sadar MD, 2017. Non-Genomic Actions of the Androgen Receptor in Prostate Cancer. Front. Endocrinol 10.3389/fendo.2017.00002. PMC5239799 28144231

[R154] Levin ER, 2005. Integration of the extranuclear and nuclear actions of estrogen. Mol Endocrinol 8 10.1210/me.2004-0390. PMC1249516 15705661

[R155] Li X, Huang J, Yi P, Bambara RA, Hilf R, Muyan M, 2004. Single-chain estrogen receptors (ERs) reveal that the ERalpha/beta heterodimer emulates functions of the ERalpha dimer in genomic estrogen signaling pathways. Mol Cell Biol 17 10.1128/MCB.24.17.7681-7694.2004. PMC506997 15314175

[R156] Liao RS, Ma S, Miao L, Li R, Yin Y, Raj GV, 2013. Androgen receptor-mediated non-genomic regulation of prostate cancer cell proliferation. Transl Androl Urol 3 10.3978/j.issn.2223-4683.2013.09.07. PMC4708176 26816736

[R157] Lin HY, Xu Q, Yeh S, Wang RS, Sparks JD, Chang C, 2005. Insulin and leptin resistance with hyperleptinemia in mice lacking androgen receptor. Diabetes 6. 10.2337/diabetes.54.6.1717. 15919793

[R158] Liu JYH, Lin G, Fang M, Rudd JA, 2019a. Localization of estrogen receptor ERalpha, ERbeta and GPR30 on myenteric neurons of the gastrointestinal tract and their role in motility. Gen Comp Endocrinol 10.1016/j.ygcen.2018.11.016. 30502347

[R159] Liu JYH, Lin G, Fang M, Rudd JA, 2019b. Localization of estrogen receptor ERα, ERβ and GPR30 on myenteric neurons of the gastrointestinal tract and their role in motility. Gen. Comp. Endocrinol 10.1016/j.ygcen.2018.11.016. 30502347

[R160] Lu ML, Schneider MC, Zheng Y, Zhang X, Richie JP, 2001. Caveolin-1 interacts with androgen receptor A positive modulator of androgen receptor mediated transactivation. J. Biol. Chem 16, doi. 10.1074/jbc.M006598200 11278309

[R161] Lu Q, Pallas DC, Surks HK, Baur WE, Mendelsohn ME, Karas RH, 2004. Striatin assembles a membrane signaling complex necessary for rapid, nongenomic activation of endothelial NO synthase by estrogen receptor alpha. Proc Natl Acad Sci U S A 49 10.1073/pnas.0407492101. PMC534607 15569929

[R162] Lucas-Herald AK, Alves-Lopes R, Montezano AC, Ahmed SF, and Touyz RM, 2017. Genomic and non-genomic effects of androgens in the cardiovascular system: clinical implications. Clinical science (London, England : 1979) 13, doi: 10.1042/CS20170090. PMC5736922 28645930

[R163] Lund TD, Salyer DL, Fleming DE, Lephart ED, 2000. Pre- or postnatal testosterone and flutamide effects on sexually dimorphic nuclei of the rat hypothalamus. Dev. Brain Res 2 10.1016/S0165-3806(00)00013-4. 10775778

[R164] Lutz LB, Jamnongjit M, Yang WH, Jahani D, Gill A, Hammes SR, 2003. Selective modulation of genomic and nongenomic androgen responses by androgen receptor ligands. Mol Endocrinol 6 10.1210/me.2003-0032. 12637588

[R165] MacLusky NJ, Hajszan T, Prange-Kiel J, Leranth C, 2006. Androgen modulation of hippocampal synaptic plasticity. Neuroscience 3. 10.1016/j.neuroscience.2005.12.054. 16488544

[R166] Maggioli E, McArthur S, Mauro C, Kieswich J, Kusters DHM, Reutelingsperger CPM, Yaqoob M, Solito E, 2016. Estrogen protects the blood–brain barrier from inflammation-induced disruption and increased lymphocyte trafficking. Brain Behav. Immun 10.1016/j.bbi.2015.08.020. 26321046

[R167] Matsumoto A, Arai Y, 1976. Effect of estrogen of early postnatal development of synaptic formation in the hypothalamic arcuate nucleus of female rats. Neurosci. Lett 2 10.1016/0304-3940(76)90027-6. 19604819

[R168] McCarthy MM, 2008. Estradiol and the developing brain. Physiol. Rev 1 10.1152/physrev.00010.2007. PMC2754262 18195084

[R169] McCulloch DR, Harvey M, Herington AC, 2000. The expression of the ADAMs proteases in prostate cancer cell lines and their regulation by dihydrotestosterone. Mol Cell Endocrinol 1–2 10.1016/s0303-7207(00)00305-1. 11000516

[R170] McEwen BS, Milner TA, 2017. Understanding the broad influence of sex hormones and sex differences in the brain. J Neurosci Res 1–2 10.1002/jnr.23809. PMC5120618 27870427

[R171] McGinnis MY, Williams GW, Lumia AR, 1996. Inhibition of male sex behavior by androgen receptor blockade in preoptic area or hypothalamus, but not amygdala or septum. Physiol. Behav 3 10.1016/0031-9384(96)00088-1. 8873251

[R172] Menazza S, Sun J, Appachi S, Chambliss KL, Kim SH, Aponte A, Khan S, Katzenellenbogen JA, Katzenellenbogen BS, Shaul PW, Murphy E, 2017. Non-nuclear estrogen receptor alpha activation in endothelium reduces cardiac ischemia-reperfusion injury in mice. J Mol Cell Cardiol 10.1016/j.yjmcc.2017.04.004. PMC5514412 28457941

[R173] Métivier R, Penot G, Hübner MR, Reid G, Brand H, Koš M, Gannon F, 2003. Estrogen receptor-α directs ordered, cyclical, and combinatorial recruitment of cofactors on a natural target promoter. Cell 6, doi. 10.1016/s0092-8674(03)00934-6 14675539

[R174] Meyer MR, Amann K, Field AS, Hu C, Hathaway HJ, Kanagy NL, Walker MK, Barton M, Prossnitz ER, 2012. Deletion of G protein-coupled estrogen receptor increases endothelial vasoconstriction. Hypertension 2. 10.1161/HYPERTENSIONAHA.111.184606. PMC3266468 22203741

[R175] Mhaouty-Kodja S, 2017. Role of the androgen receptor in the central nervous system. Mol. Cell. Endocrinol 10.1016/j.mce.2017.08.001. 28826929

[R176] McHenry J, Carrier N, Hull E, Kabbaj M, 2014. Sex differences in anxiety and depression: role of testosterone. Front. Neuroendocrinol 1 10.1016/j.yfrne.2013.09.001. PMC3946856 24076484

[R177] Milner TA, Ayoola K, Drake CT, Herrick SP, Tabori NE, McEwen BS, Warrier S, Alves SE, 2005. Ultrastructural localization of estrogen receptor β immunoreactivity in the rat hippocampal formation. Journal of Comparative Neurology 2 10.1002/cne.20724. 16127691

[R178] Milner TA, McEwen BS, Hayashi S, Li CJ, Reagan LP, Alves SE, 2001. Ultrastructural evidence that hippocampal alpha estrogen receptors are located at extranuclear sites. Journal of Comparative Neurology 3 10.1002/1096-9861(20010115)429:3<355::AID-CNE1>3.0.CO;2-#. 11116225

[R179] Miranda A, Sousa N, 2018. Maternal hormonal milieu influence on fetal brain development. Brain and behavior 2 10.1002/brb3.920. PMC5822586 29484271

[R180] Mitsushima D, Takase K, Funabashi T, Kimura F, 2009a. Gonadal steroids maintain 24 h acetylcholine release in the hippocampus: organizational and activational effects in behaving rats. J Neurosci 12 10.1523/JNEUROSCI.5301-08.2009. PMC6665029 19321777

[R181] Mitsushima D, Takase K, Takahashi T, Kimura F, 2009b. Activational and organisational effects of gonadal steroids on sex-specific acetylcholine release in the dorsal hippocampus. J Neuroendocrinol 4 10.1111/j.1365-2826.2009.01848.x. 19356199

[R182] Mitterling KL, Spencer JL, Dziedzic N, Shenoy S, McCarthy K, Waters EM, McEwen BS, Milner TA, 2010. Cellular and subcellular localization of estrogen and progestin receptor immunoreactivities in the mouse hippocampus. J. Comp. Neurol 14 10.1002/cne.22361. PMC2879091 20506473

[R183] Mizutani T, Nishikawa Y, Adachi H, Enomoto T, Ikegami H, Kurachi H, Nomura T, Miyake A, 1994. Identification of estrogen receptor in human adipose tissue and adipocytes. The Journal of Clinical Endocrinology & Metabolism 4, doi. 10.1210/jcem.78.4.8157726 8157726

[R184] Moghadami S, Jahanshahi M, Sepehri H, Amini H, 2016. Gonadectomy reduces the density of androgen receptor-immunoreactive neurons in male rat’s hippocampus: testosterone replacement compensates it. Behavioral and Brain Functions 1 10.1186/s12993-016-0089-9. PMC4730763 26822779

[R185] Montelli S, Suman M, Corain L, Cozzi B, Peruffo A, 2017. Sexually Diergic Trophic Effects of Estradiol Exposure on Developing Bovine Cerebellar Granule Cells. Neuroendocrinology 1. 10.1159/000444528. 26882349

[R186] Morley P, Whitfield JF, Vanderhyden BC, Tsang BK, Schwartz JL, 1992. A new, nongenomic estrogen action: the rapid release of intracellular calcium. Endocrinology 3. 10.1210/endo.131.3.1505465. 1505465

[R187] Morrell MJ, Flynn KL, Done S, Flaster E, Kalayjian L, Pack AM, 2005. Sexual dysfunction, sex steroid hormone abnormalities, and depression in women with epilepsy treated with antiepileptic drugs. Epilepsy Behav 3 10.1016/j.yebeh.2005.01.004. 15820344

[R188] Morton RW, Sato K, Gallaugher MPB, Oikawa SY, McNicholas PD, Fujita S, and Phillips SM, 2018. Muscle Androgen Receptor Content but Not Systemic Hormones Is Associated With Resistance Training-Induced Skeletal Muscle Hypertrophy in Healthy, Young Men. Front Physiol doi: 10.3389/fphys.2018.01373. PMC6189473 30356739

[R189] Namwanje M, Brown CW, 2016. Activins and Inhibins: Roles in Development, Physiology, and Disease. Cold Spring Harb Perspect Biol 7 10.1101/cshperspect.a021881. PMC4930927 27328872

[R190] Nguyen T-V, McCracken JT, Albaugh MD, Botteron KN, Hudziak JJ, Ducharme S, 2016. A testosterone-related structural brain phenotype predicts aggressive behavior from childhood to adulthood. Psychoneuroendocrinology 10.1016/j.psyneuen.2015.09.021. PMC4695305 26431805

[R191] Nguyen TV, 2018. Developmental effects of androgens in the human brain. J. Neuroendocrinol 2 10.1111/jne.12486. 28489322

[R192] Niranjan MK, Srivastava R, 2019. Expression of estrogen receptor alpha in developing brain, ovary and shell gland of Gallus gallus domesticus: Impact of stress and estrogen. Steroids 10.1016/j.steroids.2019.03.002. 30885650

[R193] Norman AW, Henry HL, Bishop JE, Song X-D, Bula C, and Okamura WH, 2001. Different shapes of the steroid hormone 1α,25(OH)2-vitamin D3 act as agonists for two different receptors in the vitamin D endocrine system to mediate genomic and rapid responses☆11☆ Guest Editor: Dr. Satya Reddy, Proceedings of the First International Conference on Chemistry and Biology of Vitamin D Analogs, Brown University, Providence, RI. Steroids 3, doi: 10.1016/S0039-128X(00)00165-3. 11179722

[R194] Norman AW, Mizwicki MT, Norman DPG, 2004. Steroid-hormone rapid actions, membrane receptors and a conformational ensemble model. Nat. Rev. Drug Discovery 1. 10.1038/nrd1283. 14708019

[R195] O’Keefe JA, Handa RJ, 1990. Transient elevation of estrogen receptors in the neonatal rat hippocampus. Dev. Brain Res 1 10.1016/0165-3806(90)90191-Z. 2090365

[R196] Ogola BO, Zimmerman MA, Sure VN, Gentry KM, Duong JL, Clark GL, Miller KS, Katakam PVG, Lindsey SH, 2019. G Protein-Coupled Estrogen Receptor Protects From Angiotensin II-Induced Increases in Pulse Pressure and Oxidative Stress. Front Endocrinol (Lausanne) 10.3389/fendo.2019.00586. PMC6718465 31507536

[R197] Okamoto M, Hojo Y, Inoue K, Matsui T, Kawato S, McEwen BS, Soya H, 2012. Mild exercise increases dihydrotestosterone in hippocampus providing evidence for androgenic mediation of neurogenesis. Proc. Natl. Acad. Sci 32 10.1073/pnas.1210023109. PMC3420174 22807478

[R198] Olde B, and Lee J, 2009. GPR30/GPER1: Searching for a role in estrogen physiology. Trends in endocrinology and metabolism: TEM doi: 10.1016/j.tem.2009.04.006. 19734054

[R199] Pandini G, Mineo R, Frasca F, Roberts CT, Marcelli M, Vigneri R, Belfiore A, 2005. Androgens Up-regulate the Insulin-like Growth Factor-I Receptor in Prostate Cancer Cells. Cancer Res 5 10.1158/0008-5472.CAN-04-1837. 15753383

[R200] Panizzon MS, Hauger RL, Xian H, Jacobson K, Lyons MJ, Franz CE, Kremen WS, 2018. Interactive effects of testosterone and cortisol on hippocampal volume and episodic memory in middle-aged men. Psychoneuroendocrinology 10.1016/j.psyneuen.2018.03.003. PMC5931212 29547742

[R201] Papakonstanti EA, Kampa M, Castanas E, Stournaras C, 2003. A rapid, nongenomic, signaling pathway regulates the actin reorganization induced by activation of membrane testosterone receptors. Mol Endocrinol 5 10.1210/me.2002-0253. 12554777

[R202] Pedram A, Razandi M, Lubahn D, Liu J, Vannan M, Levin ER, 2008. Estrogen inhibits cardiac hypertrophy: role of estrogen receptor-beta to inhibit calcineurin. Endocrinology 7. 10.1210/en.2008-0133. PMC2453079 18372323

[R203] Pedram A, Razandi M, Sainson R, Kim J, Hughes C, Levin E, 2007. A Conserved Mechanism for Steroid Receptor Translocation to the Plasma Membrane. The Journal of biological chemistry 10.1074/jbc.M611877200. 17535799

[R204] Peper JS, Koolschijn PCM, Crone EA, 2013. Development of risk taking: contributions from adolescent testosterone and the orbito-frontal cortex. J. Cognit. Neurosci 12, doi. 10.1162/jocn_a_00445 23859649

[R205] Perez-Pouchoulen M, Miquel M, Saft P, Brug B, Toledo R, Hernandez ME, Manzo J, 2016a. Prenatal exposure to sodium valproate alters androgen receptor expression in the developing cerebellum in a region and age specific manner in male and female rats. Int. J. Dev. Neurosci 10.1016/j.ijdevneu.2016.07.001. 27423376

[R206] Perez-Pouchoulen M, Toledo R, Garcia LI, Perez-Estudillo CA, Coria-Avila GA, Hernandez ME, Carrillo P, Manzo J, 2016b. Androgen receptors in Purkinje neurons are modulated by systemic testosterone and sexual training in a region-specific manner in the male rat. Physiol. Behav 10.1016/j.physbeh.2016.01.027. 26812590

[R207] Perez-Pouchoulen M, Yu SJ, Roby CR, Bonsavage N, McCarthy MM, 2019. Regulatory Control of Microglial Phagocytosis by Estradiol and Prostaglandin E2 in the Developing Rat Cerebellum. The Cerebellum 5 10.1007/s12311-019-01071-z. PMC6763383 31435854

[R208] Pérez SE, Chen EY, Mufson EJ, 2003. Distribution of estrogen receptor alpha and beta immunoreactive profiles in the postnatal rat brain. Dev. Brain Res 1 10.1016/S0165-3806(03)00223-2. 14519499

[R209] Phan A, Lancaster K, Armstrong J, Maclusky N, Choleris E, 2011. Rapid Effects of Estrogen Receptor α and β Selective Agonists on Learning and Dendritic Spines in Female Mice. Endocrinology 10.1210/en.2010-1273. 21285321

[R210] Piekarski DJ, Boivin JR, Wilbrecht L, 2017. Ovarian Hormones Organize the Maturation of Inhibitory Neurotransmission in the Frontal Cortex at Puberty Onset in Female Mice. Curr. Biol 12 10.1016/j.cub.2017.05.027. PMC5699709 28578932

[R211] Pierman S, Sica M, Allieri F, Viglietti-Panzica C, Panzica GC, Bakker J, 2008. Activational effects of estradiol and dihydrotestosterone on social recognition and the arginine-vasopressin immunoreactive system in male mice lacking a functional aromatase gene. Horm Behav 1 10.1016/j.yhbeh.2008.02.001. PMC2706693 18346740

[R212] Pike CJ, Nguyen T-VV, Ramsden M, Yao M, Murphy MP, Rosario ER, 2008. Androgen cell signaling pathways involved in neuroprotective actions. Horm. Behav 5 10.1016/j.yhbeh.2007.11.006. PMC2424283 18222446

[R213] Pinares-Garcia P, Stratikopoulos M, Zagato A, Loke H, Lee J, 2018. Sex: a significant risk factor for neurodevelopmental and neurodegenerative disorders. Brain sciences 8, doi. 10.3390/brainsci8080154 PMC6120011 30104506

[R214] Plante BJ, Lessey BA, Taylor RN, Wang W, Bagchi MK, Yuan L, Scotchie J, Fritz MA, Young SL, 2012. G protein-coupled estrogen receptor (GPER) expression in normal and abnormal endometrium. Reproductive sciences (Thousand Oaks, Calif.) 7. 10.1177/1933719111431000. PMC3438071 22378861

[R215] Prescott JL, Blok L, Tindall DJ, 1998. Isolation and androgen regulation of the human homeobox cDNA, NKX3.1. Prostate 1. 10.1002/(SICI)1097-0045(19980401)35:1<71::AID-PROS10>3.0.CO;2-H. 9537602

[R216] Qiu Y, Gao Y, Yu D, Zhong L, Cai W, Ji J, Geng F, Tang G, Zhang H, Cao J, Zhang J, Zhang S, 2020. Genome-Wide Analysis Reveals Zinc Transporter ZIP9 Regulated by DNA Methylation Promotes Radiation-Induced Skin Fibrosis via the TGF-beta Signaling Pathway. J Invest Dermatol 1 10.1016/j.jid.2019.04.027. 31254515

[R217] Revankar CM, Cimino DF, Sklar LA, Arterburn JB, Prossnitz ER, 2005. A transmembrane intracellular estrogen receptor mediates rapid cell signaling. Science 5715. 10.1126/science.1106943. 15705806

[R218] Roque C, Mendes-Oliveira J, Duarte-Chendo C, Baltazar G, 2019. The role of G protein-coupled estrogen receptor 1 on neurological disorders. Front Neuroendocrinol 10.1016/j.yfrne.2019.100786. 31513775

[R219] Roselli CE, Horton LE, Resko JA, 1985. Distribution and Regulation of Aromatase Activity in the Rat Hypothalamus and Limbic System*. Endocrinology 6. 10.1210/endo-117-6-2471. 4065042

[R220] Roy AK, Lavrovsky Y, Song CS, Chen S, Jung MH, Velu NK, Bi BY, Chatterjee B, 1999. Regulation of androgen action. Vitam Horm 10.1016/s0083-6729(08)60938-3. 9949684

[R221] Ruiz D, Padmanabhan V, Sargis RM, 2020. Stress, Sex, and Sugar: Glucocorticoids and Sex-Steroid Crosstalk in the Sex-Specific Misprogramming of Metabolism. J. Endocr. Soc 8 10.1210/jendso/bvaa087. PMC7382384 32734132

[R222] Sandner F, Welter H, Schwarzer JU, Köhn FM, Urbanski HF, Mayerhofer A, 2014. Expression of the oestrogen receptor GPER by testicular peritubular cells is linked to sexual maturation and male fertility. Andrology 5 10.1111/j.2047-2927.2014.00243.x. PMC4134690 25052196

[R223] Santos RS, Frank AP, Fatima LA, Palmer BF, Oz OK, Clegg DJ, 2018. Activation of estrogen receptor alpha induces beiging of adipocytes. Mol Metab 10.1016/j.molmet.2018.09.002. PMC6309577 30270132

[R224] Sárvári M, Kalló I, Hrabovszky E, Solymosi N, Rodolosse A, Liposits Z, 2016. Long-Term Estrogen Receptor Beta Agonist Treatment Modifies the Hippocampal Transcriptome in Middle-Aged Ovariectomized Rats. Front. Cell. Neurosci 149 10.3389/fncel.2016.00149. PMC4901073 27375434

[R225] Sato T, Matsumoto T, Kawano H, Watanabe T, Uematsu Y, Sekine K, Fukuda T, Aihara K, Krust A, Yamada T, Nakamichi Y, Yamamoto Y, Nakamura T, Yoshimura K, Yoshizawa T, Metzger D, Chambon P, Kato S, 2004. Brain masculinization requires androgen receptor function. Proc Natl Acad Sci U S A 6 10.1073/pnas.0305303101. PMC341816 14747651

[R226] Scaling AL, Prossnitz ER, Hathaway HJ, 2014. GPER mediates estrogen-induced signaling and proliferation in human breast epithelial cells and normal and malignant breast. Horm Cancer 3 10.1007/s12672-014-0174-1. PMC4091989 24718936

[R227] Shanmugan S, Epperson CN, 2014. Estrogen and the prefrontal cortex: towards a new understanding of estrogen’s effects on executive functions in the menopause transition. Hum. Brain Mapp 3 10.1002/hbm.22218. PMC4104582 23238908

[R228] Shen L, Wang DQH, Lo C-M, Tso P, Davidson WS, Woods SC, Liu M, 2010. Estradiol increases the anorectic effect of central apolipoprotein A-IV. Endocrinology 7. 10.1210/en.2010-0203. PMC2903939 20484461

[R229] Sheppard KM, Padmanabhan V, Coolen LM, Lehman MN, 2011. Prenatal Programming by Testosterone of Hypothalamic Metabolic Control Neurones in the Ewe. J. Neuroendocrinol 5 10.1111/j.1365-2826.2011.02126.x. PMC3939689 21418339

[R230] Sheppard PAS, Koss WA, Frick KM, Choleris E, 2018. Rapid actions of oestrogens and their receptors on memory acquisition and consolidation in females. J. Neuroendocrinol 2 10.1111/jne.12485. PMC6543823 28489296

[R231] Schams D, 2003. Expression and localisation of oestrogen and progesterone receptors in the bovine mammary gland during development, function and involution. J. Endocrinol 10.1677/joe.0.1770305. 12740019

[R232] Schlegel A, Wang CG, Katzenellenbogen B, Pestell R, and Lisanti M, 1999. Caveolin-1 potentiates estrogen receptor alpha (ERalpha) signaling. caveolin-1 drives ligand-independent nuclear translocation and activation of ERalpha. The Journal of biological chemistry doi: 10.1074/jbc.274.47.33551. 10559241

[R233] Schulz KM, Sisk CL, 2016. The organizing actions of adolescent gonadal steroid hormones on brain and behavioral development. Neurosci. Biobehav. Rev 10.1016/j.neubiorev.2016.07.036. PMC5074860 27497718

[R234] Schutter DJLG, Meuwese R, Bos MGN, Crone EA, Peper JS, 2017. Exploring the role of testosterone in the cerebellum link to neuroticism: From adolescence to early adulthood. Psychoneuroendocrinology 10.1016/j.psyneuen.2017.01.009. 28214680

[R235] Schwartz N, Verma A, Bivens CB, Schwartz Z, Boyan BD, 2016. Rapid steroid hormone actions via membrane receptors. Biochimica et Biophysica Acta (BBA) - Molecular. Cell Res 9 10.1016/j.bbamcr.2016.06.004. 27288742

[R236] Smeeth DM, Kourouzidou I, Duarte RRR, Powell TR, Thuret S, 2020. Prolactin, Estradiol and Testosterone Differentially Impact Human Hippocampal Neurogenesis in an In Vitro Model. Neuroscience 10.1016/j.neuroscience.2019.12.021. PMC7839971 31930958

[R237] Soltysik K, and Czekaj P, 2013. MEmbrane estrogen receptors - Is it an alternative way of estrogen action? Journal of physiology and pharmacology : an official journal of the Polish Physiological Society 23756388

[R238] Solum DT, Handa RJ, 2001. Localization of estrogen receptor alpha (ERα) in pyramidal neurons of the developing rat hippocampus. Dev. Brain Res 2 10.1016/S0165-3806(01)00171-7. 11412902

[R239] Solum DT, Handa RJ, 2002. Estrogen regulates the development of brain-derived neurotrophic factor mRNA and protein in the rat hippocampus. J. Neurosci 7, doi. 10.1523/JNEUROSCI.22-07-02650.2002 PMC6758321 11923430

[R240] Sørvik IB, Paulsen RE, 2017. High and low concentration of 17α-estradiol protect cerebellar granule neurons in different time windows. Biochem. Biophys. Res. Commun 3 10.1016/j.bbrc.2017.06.100. 28634070

[R241] Sperry TS, Thomas P, 1999. Characterization of two nuclear androgen receptors in Atlantic croaker: comparison of their biochemical properties and binding specificities. Endocrinology 4. 10.1210/endo.140.4.6631. 10098494

[R242] Spritzer MD, Roy EA, 2020. Testosterone and Adult Neurogenesis. Biomolecules 2 10.3390/biom10020225. PMC7072323 32028656

[R243] Stewart J, Rajabi H, 1994. Estradiol derived from testosterone in prenatal life affects the development of catecholamine systems in the frontal cortex in the male rat. Brain Res 1 10.1016/0006-8993(94)90070-1. 8055334

[R244] Sukocheva O, Li B, Due S, Hussey D, and Watson D, 2015. Androgens and esophageal cancer: What do we know? World journal of gastroenterology : WJG doi: 10.3748/wjg.v21.i20.6146. PMC4445092 26034350

[R245] Sun T, Liu Z, Liu M, Guo Y, Sun H, Zhao J, Lan Z, Lian B, Zhang J, 2019. Hippocampus-specific Rictor knockdown inhibited 17beta-estradiol induced neuronal plasticity and spatial memory improvement in ovariectomized mice. Behav Brain Res 10.1016/j.bbr.2019.02.014. 30753873

[R246] Swaab DF, and Garcia-Falgueras A, 2009. Sexual differentiation of the human brain in relation to gender identity and sexual orientation. Functional neurology 19403051

[R247] Taniguchi M, Fukunaka A, Hagihara M, Watanabe K, Kamino S, Kambe T, Enomoto S, Hiromura M, 2013. Essential role of the zinc transporter ZIP9/SLC39A9 in regulating the activations of Akt and Erk in B-cell receptor signaling pathway in DT40 cells. PLoS ONE 3. 10.1371/journal.pone.0058022. PMC3591455 23505453

[R248] Thomas P, Converse A, Berg H, 2017a. ZIP9, a novel membrane androgen receptor and zinc transporter protein. Gen. Comp. Endocrinol 10.1016/j.ygcen.2017.04.016. 28479083

[R249] Thomas P, Pang Y, Dong J, 2017b. Membrane androgen receptor characteristics of human ZIP9 (SLC39A) zinc transporter in prostate cancer cells: Androgen-specific activation and involvement of an inhibitory G protein in zinc and MAP kinase signaling. Mol. Cell. Endocrinol 10.1016/j.mce.2017.02.025. 28219737

[R250] Thomas P, Pang Y, Dong J, Berg AH, 2014. Identification and characterization of membrane androgen receptors in the ZIP9 zinc transporter subfamily: II. Role of human ZIP9 in testosterone-induced prostate and breast cancer cell apoptosis. Endocrinology 11. 10.1210/en.2014-1201. PMC4197988 25014355

[R251] Tobiansky DJ, Wallin-Miller KG, Floresco SB, Wood RI, Soma KK, 2018a. Androgen Regulation of the Mesocorticolimbic System and Executive Function. Front. Endocrinol 10.3389/fendo.2018.00279. PMC5996102 29922228

[R252] Tobiansky DJ, Wallin-Miller KG, Floresco SB, Wood RI, Soma KK, 2018b. Androgen Regulation of the Mesocorticolimbic System and Executive Function. Front. Endocrinol 279 10.3389/fendo.2018.00279. PMC5996102 29922228

[R253] Tonn Eisinger KR, Larson EB, Boulware MI, Thomas MJ, Mermelstein PG, 2018. Membrane estrogen receptor signaling impacts the reward circuitry of the female brain to influence motivated behaviors. Steroids 10.1016/j.steroids.2017.11.013. PMC5864533 29195840

[R254] Tsutsui K, 2012. Neurosteroid biosynthesis and action during cerebellar development. The Cerebellum 2, doi. 10.1007/s12311-011-0341-7 22198872

[R255] Tu J, Jufri NF, 2013. Estrogen signaling through estrogen receptor beta and G-protein-coupled estrogen receptor 1 in human cerebral vascular endothelial cells: implications for cerebral aneurysms. Biomed Res. Int 10.1155/2013/524324. PMC3844273 24319683

[R256] Tuscher JJ, Szinte JS, Starrett JR, Krentzel AA, Fortress AM, Remage-Healey L, Frick KM, 2016. Inhibition of local estrogen synthesis in the hippocampus impairs hippocampal memory consolidation in ovariectomized female mice. Horm Behav 10.1016/j.yhbeh.2016.05.001. PMC4915975 27178577

[R257] Tuscher JJ, Taxier LR, Fortress AM, Frick KM, 2018. Chemogenetic inactivation of the dorsal hippocampus and medial prefrontal cortex, individually and concurrently, impairs object recognition and spatial memory consolidation in female mice. Neurobiol. Learn. Mem 10.1016/j.nlm.2018.11.002. PMC7310386 30408525

[R258] Tuscher JJ, Taxier LR, Schalk JC, Haertel JM, Frick KM, 2019. Chemogenetic Suppression of Medial Prefrontal-Dorsal Hippocampal Interactions Prevents Estrogenic Enhancement of Memory Consolidation in Female Mice. eNeuro 2 10.1523/ENEURO.0451-18.2019. PMC6477593 31016230

[R259] Tyborowska A, Volman I, Smeekens S, Toni I, Roelofs K, 2016. Testosterone during Puberty Shifts Emotional Control from Pulvinar to Anterior Prefrontal Cortex. The Journal of Neuroscience 23 10.1523/JNEUROSCI.3874-15.2016. PMC6604884 27277794

[R260] Vadakkadath Meethal S, Atwood CS, 2005. The role of hypothalamic-pituitary-gonadal hormones in the normal structure and functioning of the brain. Cell Mol Life Sci 3 10.1007/s00018-004-4381-3. PMC11924492 15723162

[R261] Varshney M, Nalvarte I, 2017. Genes, Gender, Environment, and Novel Functions of Estrogen Receptor Beta in the Susceptibility to Neurodevelopmental Disorders. Brain sciences 3 10.3390/brainsci7030024. PMC5366823 28241485

[R262] Varshney MK, Inzunza J, Lupu D, Ganapathy V, Antonson P, Ruegg J, Nalvarte I, Gustafsson JA, 2017. Role of estrogen receptor beta in neural differentiation of mouse embryonic stem cells. Proc Natl Acad Sci U S A 48 10.1073/pnas.1714094114. PMC5715781 29133394

[R263] Vidal O, Lindberg M, Sävendahl L, Lubahn DB, Ritzen EM, Gustafsson JÅ, Ohlsson C, 1999. Disproportional Body Growth in Female Estrogen Receptor-α-Inactivated Mice. Biochem. Biophys. Res. Commun 2 10.1006/bbrc.1999.1711. 10558910

[R264] Vigil P, Del Río JP, Carrera B, ArÁnguiz FC, Rioseco H, Cortés ME, 2016. Influence of sex steroid hormones on the adolescent brain and behavior: An update. Linacre Q 3 10.1080/00243639.2016.1211863. PMC5102198 27833209

[R265] Volman I, von Borries AKL, Bulten BH, Verkes RJ, Toni I, and Roelofs K, 2016. Testosterone Modulates Altered Prefrontal Control of Emotional Actions in Psychopathic Offenders(1,2,3). eNeuro 1, doi: 10.1523/ENEURO.0107-15.2016. PMC4745181 26878057

[R266] Walters MR, Hunziker W, Norman AW, 1981. A mathematical model describing the subcellular localization of non-membrane bound steroid, seco-steroid and thyronine receptors. J. Steroid Biochem 10.1016/0022-4731(81)90320-4. 6279971

[R267] Wang H, Jessup JA, Lin MS, Chagas C, Lindsey SH, Groban L, 2012. Activation of GPR30 attenuates diastolic dysfunction and left ventricle remodelling in oophorectomized mRen2.Lewis rats. Cardiovasc Res 1 10.1093/cvr/cvs090. PMC3307382 22328091

[R268] Watanabe T, Inoue S, Hiroi H, Orimo A, Kawashima H, Muramatsu M, 1998. Isolation of estrogen-responsive genes with a CpG island library. Mol. Cell. Biol 1 10.1128/mcb.18.1.442. PMC121513 9418891

[R269] Waters EM, Simerly RB, 2009. Estrogen Induces Caspase-Dependent Cell Death during Hypothalamic Development. The Journal of Neuroscience 31 10.1523/JNEUROSCI.0135-09.2009. PMC6428191 19657024

[R270] Weige CC, Allred KF, Armstrong CM, Allred CD, 2012. P53 mediates estradiol induced activation of apoptosis and DNA repair in non-malignant colonocytes. The Journal of steroid biochemistry and molecular biology 3–5, doi. 10.1016/j.jsbmb.2011.10.010 22100717

[R271] Welboren W-J, Stunnenberg HG, Sweep FCGJ, Span PN, 2007. Identifying estrogen receptor target genes. Mol. Oncol 2 10.1016/j.molonc.2007.04.001. PMC5543883 19383291

[R272] Wierenga LM, Bos MG, Schreuders E, vd Kamp F, Peper JS, Tamnes CK, and Crone EA, 2018. Unraveling age, puberty and testosterone effects on subcortical brain development across adolescence. Psychoneuroendocrinology10.1016/j.psyneuen.2018.02.034 29547741

[R273] Williams C, DiLeo A, Niv Y, Gustafsson J-Å, 2016. Estrogen receptor beta as target for colorectal cancer prevention. Cancer Lett 1 10.1016/j.canlet.2015.12.009. PMC4744541 26708506

[R274] Williams CL, 1986. A reevaluation of the concept of separable periods of organizational and activational actions of estrogens in development of brain and behavior. Annals of the New York Academy of Sciences10.1111/j.1749-6632.1986.tb28019.x 3555228

[R275] Wright CL, Hoffman JH, McCarthy MM, 2019. Evidence that inflammation promotes estradiol synthesis in human cerebellum during early childhood. Transl. Psychiatry 1. 10.1038/s41398-018-0363-8. PMC6355799 30705253

[R276] Xu Y, Sheng H, Bao Q, Wang Y, Lu J, Ni X, 2016. NLRP3 inflammasome activation mediates estrogen deficiency-induced depression- and anxiety-like behavior and hippocampal inflammation in mice. Brain Behav. Immun 10.1016/j.bbi.2016.02.022. 26928197

[R277] Xu Z, Liu J, Gu L, Huang B, Pan X, 2017. Biological effects of xenoestrogens and the functional mechanisms via genomic and nongenomic pathways. Environmental Reviews 10.1139/er-2016-0075.

[R278] Yuen EY, Wei J, Yan Z, 2016. Estrogen in prefrontal cortex blocks stress-induced cognitive impairments in female rats. The Journal of Steroid Biochemistry and Molecular Biology 10.1016/j.jsbmb.2015.08.028. PMC4769981 26321384

[R279] Zhang B, Kwon O-J, Henry G, Malewska A, Wei X, Zhang L, Brinkley W, Zhang Y, Castro PD, Titus M, Chen R, Sayeeduddin M, Raj GV, Mauck R, Roehrborn C, Creighton CJ, Strand DW, Ittmann MM, Xin L, 2016. Non-Cell-Autonomous Regulation of Prostate Epithelial Homeostasis by Androgen Receptor. Mol. Cell 6. 10.1016/j.molcel.2016.07.025. PMC5026614 27594448

[R280] Zhang J-M, Konkle ATM, Zup SL, McCarthy MM, 2008. Impact of sex and hormones on new cells in the developing rat hippocampus: a novel source of sex dimorphism? The European journal of neuroscience 4 10.1111/j.1460-9568.2008.06073.x. PMC2735768 18333959

[R281] Zhao L, Huang S, Mei S, Yang Z, Xu L, Zhou N, Yang Q, Shen Q, Wang W, Le X, Lau WB, Lau B, Wang X, Yi T, Zhao X, Wei Y, Warner M, Gustafsson JA, Zhou S, 2018. Pharmacological activation of estrogen receptor beta augments innate immunity to suppress cancer metastasis. Proc Natl Acad Sci U S A 16 10.1073/pnas.1803291115. PMC5910874 29592953

[R282] Zhu BT, Han GZ, Shim JY, Wen Y, Jiang XR, 2006. Quantitative structure-activity relationship of various endogenous estrogen metabolites for human estrogen receptor alpha and beta subtypes: Insights into the structural determinants favoring a differential subtype binding. Endocrinology 9. 10.1210/en.2006-0113. 16728493

[R283] Zhu L, Shi J, Luu TN, Neuman JC, Trefts E, Yu S, Palmisano BT, Wasserman DH, Linton MF, Stafford JM, 2018. Hepatocyte estrogen receptor alpha mediates estrogen action to promote reverse cholesterol transport during Western-type diet feeding. Mol Metab 10.1016/j.molmet.2017.12.012. PMC5985047 29331506

[R284] Zinn S, Schnell M, 2018. Flexibility at the Fringes: Conformations of the Steroid Hormone β-Estradiol. ChemPhysChem 21. 10.1002/cphc.201800647. 30055108

[R285] Zuo D, Wang F, Rong W, Wen Y, Sun K, Zhao X, Ren X, He Z, Ding N, Ma L, Xu F, 2020. The novel estrogen receptor GPER1 decreases epilepsy severity and susceptivity in the hippocampus after status epilepticus. Neurosci. Lett 10.1016/j.neulet.2020.134978. 32302699

